# Rapid evolution of a voltage-gated sodium channel gene in a lineage of electric fish leads to a persistent sodium current

**DOI:** 10.1371/journal.pbio.2004892

**Published:** 2018-03-27

**Authors:** Ammon Thompson, Daniel T. Infield, Adam R. Smith, G. Troy Smith, Christopher A. Ahern, Harold H. Zakon

**Affiliations:** 1 Department of Integrative Biology, The University of Texas, Austin, Texas, United States of America; 2 Department of Neuroscience, The University of Texas, Austin, Texas, United States of America; 3 Department of Molecular Physiology and Biophysics, Iowa Neuroscience Institute, The University of Iowa, Iowa City, Iowa, United States of America; 4 Department of Biology and Center for the Integrative Study of Animal Behavior, Indiana University, Bloomington, Indiana, United States of America; University of Bath, United Kingdom of Great Britain and Northern Ireland

## Abstract

Most weakly electric fish navigate and communicate by sensing electric signals generated by their muscle-derived electric organs. Adults of one lineage (Apteronotidae), which discharge their electric organs in excess of 1 kHz, instead have an electric organ derived from the axons of specialized spinal neurons (electromotorneurons [EMNs]). EMNs fire spontaneously and are the fastest-firing neurons known. This biophysically extreme phenotype depends upon a persistent sodium current, the molecular underpinnings of which remain unknown. We show that a skeletal muscle–specific sodium channel gene duplicated in this lineage and, within approximately 2 million years, began expressing in the spinal cord, a novel site of expression for this isoform. Concurrently, amino acid replacements that cause a persistent sodium current accumulated in the regions of the channel underlying inactivation. Therefore, a novel adaptation allowing extreme neuronal firing arose from the duplication, change in expression, and rapid sequence evolution of a muscle-expressing sodium channel gene.

## Author summary

The electrical properties of excitable cells, such as those in muscle and nervous tissue, were enabled in large part by the evolution of voltage-gated ion channel genes. The regulated conduction of ions through these channels results in the propagation of electrical signals, facilitating communication between cells. Here, we investigated how voltage-gated sodium (Na_v_) channels contributed to the evolution of a novel electric organ system in the Apteronotids—a lineage of weakly electric fish. This organ is developmentally derived from motor neurons and used for communication between individual fish, as well as for probing their nocturnal environment. We used transcriptomic data to show that the gene encoding a broadly conserved muscle-specific sodium channel was duplicated in an ancestral fish. One duplicated gene copy subsequently gained expression in the spinal cord, where the electric organ is located. Through evolutionary analysis and biophysical experiments, we demonstrate that sequence changes in this new sodium channel transformed its function to cause novel electrical properties that can facilitate spontaneous high-frequency action potentials. This study shows that duplicate genes can gain highly novel expression patterns and quickly adapt to contribute to the phenotypic evolution of novel organ systems.

Electrocommunication convergently evolved within the distantly related South American and African weakly electric fishes approximately 100 million years ago (MYA) [1], enabling them to detect their environment and communicate with each other through the generation and sensation of electric signals. These species exhibit a highly derived phenotypic trait—the electric organ—which in both clades is developmentally derived from muscle cells. Electric organs have repurposed muscle action potential genes (voltage-gated ion channels) to generate the electric signal [2–4]. Electric organ signals have subsequently been shaped by both natural and sexual selection [5–10], resulting in diverse signaling patterns in both clades of electric fish [8,10].

The voltage-gated ion channels in electric organs are an ideal system for investigating the molecular mechanisms underpinning the evolution of a phenotypic trait. Voltage-gated ion channels are the primary molecular machinery producing electrical signals in excitable tissues. Well-developed electrophysiology assays make it possible to gain a mechanistic understanding of ion channels at single amino acid resolution. There is a clear and direct link between ion channel physiology and the attributes of electric organ signals that can be measured in the field and in the lab. Therefore, the diversity of electric organ signals provides an opportunity to study—in detail—natural experiments whereby ion channels evolve to directly influence a rapidly evolving phenotype.

Some species emit electric organ signals that are biophysically extreme [9,11–13]. One family within South American electric fish, the Apteronotidae, generates the highest electric organ discharge frequencies of any electric fish (depending on species, 650–1,800 Hz) [14–16]. Sustained firing rates of 1 kHz or more are difficult for neurons to attain, yet these rates are maintained continuously throughout the fish’s lifetime. Studies of two Apteronotid species (*Apteronotus albifrons* and *A*. *leptorhynchus*) illustrate the unique anatomy that underlies the family’s extreme physiology. Early in development, Apteronotids lose their muscle-derived (myogenic) electric organ [17], and the axons of neurons that innervate the electric organ (electromotorneurons [EMNs]) undergo substantial morphological transformation to form a neuronally derived (neurogenic) electric organ [18] (S1 Fig). The EMNs of *Apteronotus* are the fastest-firing neurons known [16] and can generate these signals spontaneously, independent of EMN inputs originating in the brainstem [19].

Specializations of voltage-gated ion channels likely contributed to the evolution of neurogenic electric organs found in *Apteronotus*. Spontaneously firing neurons can be driven by a persistent sodium (Na^+^) current (I_NAP_) [20]. I_NAP_ derives from sodium channels that fail to fully inactivate after each action potential—inactivation referring to the rapid closing of sodium channels, which halts the inward Na^+^ current and terminates the action potential. A persistent sodium current can cause a strong depolarization sufficient to rapidly elicit another action potential. Electrophysiological and pharmacological experiments suggest that high spontaneous rates of *Apteronotus* EMN firing are driven by I_NAP_ [20], implicating modification of voltage-gated sodium (Na_v_) channels in the production of this extreme physiology.

Gene duplication within the Na_v_ channel family appears to have played an important role in the evolution and diversification of electrocommunication and electrolocation. Early in teleost evolution, because of a whole genome duplication, the vertebrate muscle–expressing sodium channel gene (*scn4a*) duplicated into *scn4aa* and *scn4ab*, both expressed in muscle in most teleost fish [2,21]. In electric fish, *scn4aa* was independently co-opted as an electric organ–specific sodium channel in the South American and African clades, where its amino acid sequence rapidly diverged, mirroring the diversity of electric signals exhibited within each clade [3,4,22,23].

Here, we show that an additional duplication at the same muscle-expressing sodium channel locus (*scn4a*) occurred within a sublineage of Apteronotidae that includes the genus *Apteronotus*. Within approximately 2 million years, one of the resulting duplicates began expressing in the spinal cord, where the neurogenic electric organ is located. This duplicate rapidly evolved several amino acid substitutions in two structurally proximate domains of the channel that interact to mediate inactivation and that have otherwise been conserved in all vertebrate sodium channels. When combinations of these substitutions are introduced into a human sodium channel, they produce a significant I_NAP_. This is the first sodium channel that generates a physiological persistent current without the aid of auxiliary proteins [24–26]. These findings indicate two phenomena: (1) paralogs of the same muscle-type Na_v_ channel have been implicated in the evolution of three separate electrocommunication systems within teleost fishes and (2) a molecular adaptation for the extremely high firing frequencies observed in an electric organ derived from neural cells was in part mediated by a new gene that originated from the duplication of a muscle-expressing gene.

## Results

### A novel Apteronotid Na_v_ channel gene duplication

To identify Na_v_ channels expressed in the EMNs, we generated transcriptomes from the posterior spinal cords (where EMNs are abundant) and trunk muscle of adults of 3 species of Apteronotids (*A*. *albifrons*, *A*. *leptorhynchus*, and *Parapteronotus hasemani*), one myogenic electric gymnotiform (*Eigenmannia virescens)*, and a catfish (*Ictalurus punctatus*) as a nonelectric outgroup. We also obtained expression data from another myogenic gymnotiform, the electric eel (*Electrophorus electricus*), from a previous study [27].

**Fig 1:**
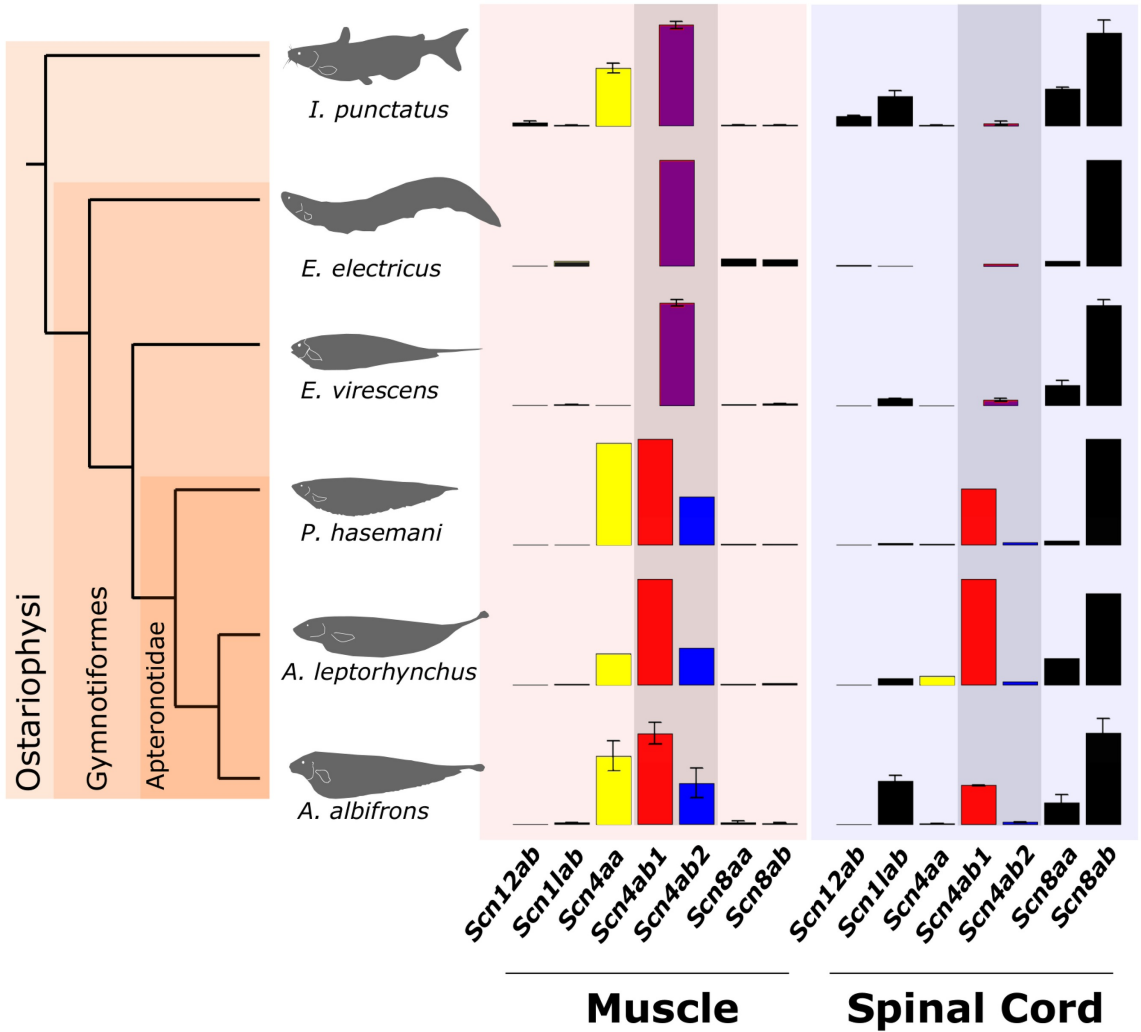
Expression of Na_v_ channel genes in muscle and spinal cord of Apteronotids (*A*. *albifrons N* = 3, *A*. *leptorhynchus N* = 1, *P*. *hasemani N* = 1), other Gymnotiforms (*E*. *virescens N* = 3, *E*. *electricus N* = 1), and the channel catfish (*I*. *punctatus N* = 3) as an outgroup, determined by NextGen sequencing. Data shown are the mean TPM of transfrags with the highest TPM for each gene. Whiskers show 1 standard deviation for species with 3 biological replicates. *scn4aa* (yellow) is expressed in muscle in most fishes except for those with myogenic electric organs, where *scn4aa* expression is lost from muscle and compartmentalized in the EO [25]. *Scn4ab* (purple) is expressed in muscle in all teleosts, including electric fish. *Scn4ab* duplicated into *scn4ab1* (red) and *scn4ab2* (blue) in the Apteronotids. *Scn4b1* expresses in muscle and spinal cord, which is a novel site of expression for the *scn4a* type gene in vertebrates. *Scn4ab2* still expresses in muscle only. The tree is a consensus tree based on Fig 2. All data points shown in S2 Fig. Figure data included in S1 Data. EO, electric organ; Na_v_, voltage-gated sodium; TPM, transcripts per million.

Data reported here confirm previous results; both *scn4a* paralogs are expressed in catfish muscle, while only *scn4ab* is present in the muscle of the myogenic electric fish *E*. *virescens* and in *E*. *electricus* (Figs 1 and S2). Unlike myogenic gymnotiforms but like nonelectric teleosts, *scn4aa* is significantly expressed in the muscle of Apteronotids (also confirmed here by quantitative PCR [qPCR], S3 Fig). We detected virtually no expression of *scn4aa* in *E*. *virescens* muscle, which confirms previous research that showed that *scn4aa* is electric organ specific and is not expressed in the muscle of myogenic gymnotiforms [2–4,27].

We also discovered a novel gene duplication in *A*. *albifrons*: 2 *scn4ab* paralogs—*scn4ab1* and *scn4ab2*—that are about 95% identical in amino acid sequence, suggesting a recent duplication event. We confirmed that these are not assembly artifacts by cloning them by PCR from muscle mRNA and amplifying them from genomic DNA across several exons (equivalent to zebrafish scn4ab exons 22–24), with the intervening introns showing even greater divergence (S4 Fig). These 2 paralogs were also found in *A*. *leptorhynchus* and *P*. *hasemani*. *Scn4ab2* showed expression in muscle, and *scn4ab1* expressed in muscle and spinal cord. In contrast to other Gymnotiform electric fish species which only express 1 subtype in muscle (*scn4ab*), these 3 Apteronotid species show significant expression of 3 sodium channels (*scn4aa*, *scn4ab1*, and *scn4ab2*).

### Rapid evolution of new Nav channel gene

In catfish, *E*. *virescens* and *E*. *electricus*, spinal cord expression is dominated by *scn8ab* and, to a lesser degree, *scn8aa* and *scn1lab*, as reported for zebrafish [21,28] and their orthologs in mammalian spinal motorneurons [29,30]. While those genes are also expressed in the Apteronotid spinal cord, *scn4ab1* makes up 21%–45% of total sodium channel expression there. The expression of *scn4ab1* in *A*. *albifrons* spinal cord was confirmed by qPCR (S3 Fig). This is the first observation of a muscle-typical Na_v_ channel gene expressing in spinal cord in any vertebrate.

**Fig 2:**
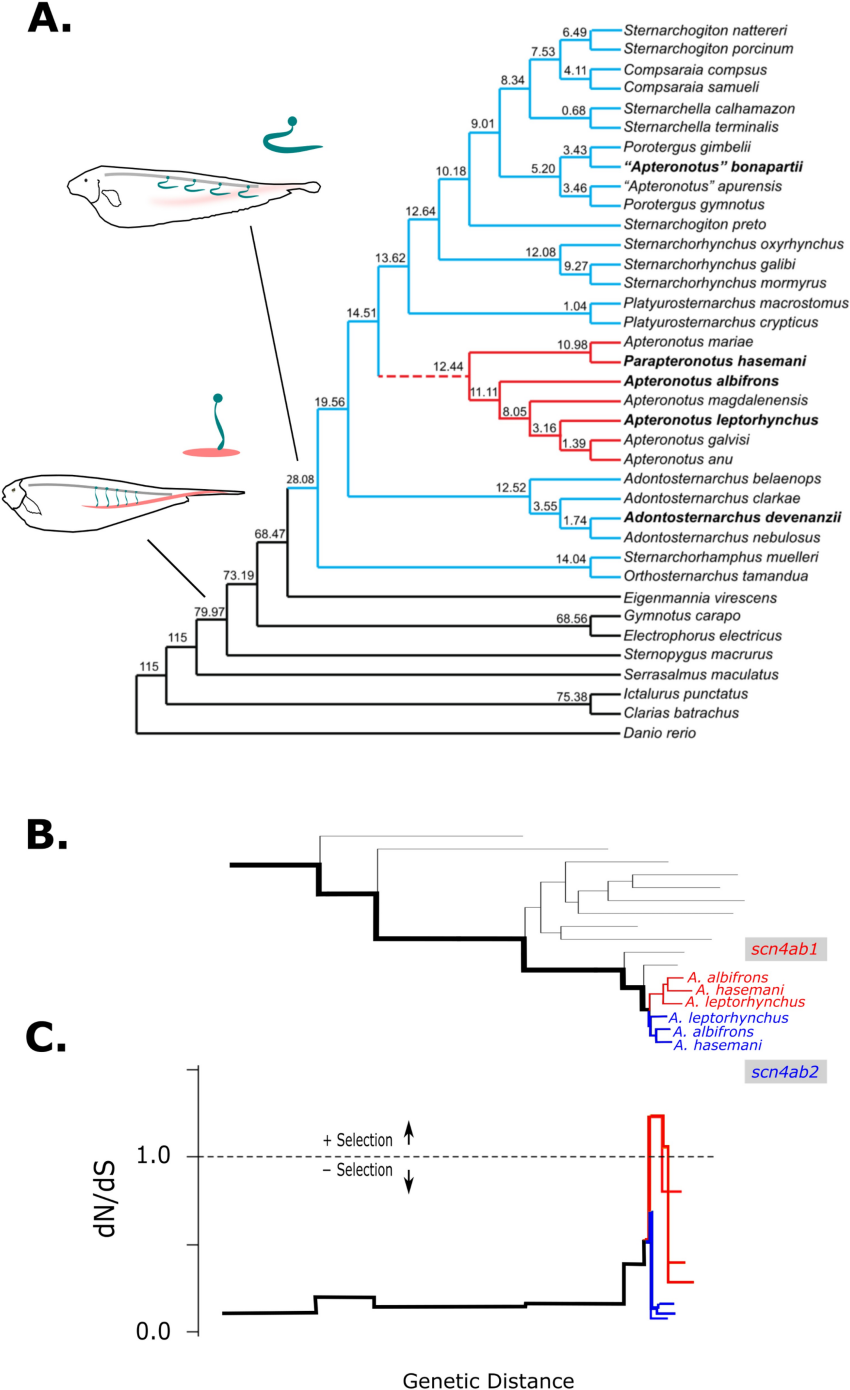
Timing of duplication and neofunctionalization of *scn4ab* paralogs. (A) Chronogram of electric species and relatives with diagrams of myogenic and neurogenic fish, and when they evolved, indicated. *Scn4ab* gene duplication and amino acid divergence occurred in a 2 MY window (dotted red line). This phylogeny is based on COI, CytB, and RAG2 genes. Molecular clock analysis shows that the *scn4ab* gene duplication occurred within the family Apteronotidae (light blue) after the last common ancestor of *A*. *devenanzi* and *“A” bonapartii* (approximately 14.5 MYA) and before the divergence of the species within the clade Apteronotini (red, approximately 12.4 MYA), which show the amino acid substitutions in the domain 4 S4–S5 linker. Species from which *scn4ab* genes have been cloned (and illustrated in S6 Fig) are in bold. Following gene duplication, *scn4ab1* shows an episodic burst of positive selection. (B) Gene tree for *scn4ab* in Gymnotiforms and closest available relatives. The red branches indicate *scn4ab1* clade and blue for *scn4ab2* clade in the 3 Apteronotini species. (C) Branchwise maximum likelihood estimates of dN/dS using the BSR method [32] from the most recent common ancestor with the zebrafish, *Danio rerio*, leading to the inferred duplication event (thick black branches in panel B). Following duplication, the *scn4ab1* clade (red) shows elevated dN/dS relative to its muscle-specific paralog (blue) in all Apteronotini species, with the branch immediately following the duplication event having a dN/dS >1. Gene accessions are in S1 Table. Figure data included in S1 Data. BSR, Branch-sites REL; COI, cytochrome c oxidase subunit I; CytB, cytochrome B; dN/dS, nonsynonymous replacements per nonsynonymous site divided by the synonymous changes per synonymous site; MY, million-year; MYA, million years ago; RAG2, recombination activating gene 2; REL, random effects likelihood.

A gene tree inferred from a nucleotide alignment of gymnotiform and non-gymnotiform *scn4ab* sodium channels with additional sequences from 2 more basal and widely separated Apteronotids (“*A*.” *bonapartii*, *Adontosternarchus devenanzi*) (S5 Fig) shows strong support for the *scn4ab1* and *scn4ab2* paralogs forming a monophyletic clade in a derived lineage within Apteronotidae called the Apteronotini [31]. A time-calibrated phylogeny of the Apteronotidae (Fig 2A) estimates the duplication of *scn4ab* at the divergence of Apteronotini from other Apteronotids at approximately 14.5 MYA (min = 5.36, max = 23.66) with most amino acid substitutions fixed by approximately 12.4 MYA (min = 3.66, max = 21.21), preceding the divergence of species within the Apteronotini. Therefore, the duplication and divergence of this gene likely occurred within an approximate 2 million–year window. Branch-specific nonsynonymous replacements per nonsynonymous site divided by the synonymous changes per synonymous site (dN/dS) ratios estimated by maximum likelihood [32] support an episodic burst of positive selection on *scn4ab1* immediately after duplication followed by an elevated dN/dS ratio within that clade (Fig 2B and 2C). The other paralog, *scn4ab2*, which shows muscle-specific expression like other vertebrate *scn4a* sodium channels, shows coding sequence patterns consistent with purifying selection, suggesting that this gene maintained its ancestral function along with ancestral expression in muscle.

We also estimated dN/dS using phylogenetic analysis by maximum likelihood (PAML) [33]. We compared a model in which the root branch of the *scn4ab1* clade following the duplication had a unique dN/dS ratio versus a model in which all branches in the tree had the same ratio. A likelihood ratio test supports the more complex model with 2 ratios, which estimates that *scn4ab1* evolved by positive selection soon after duplication (dN/dS = 1.58), while the rest of the branches in the tree show more conservative evolution with dN/dS = 0.090 (2ΔL = 38.8, df = 1, *p* < 0.0001). We were unable to find statistically significant evidence of evolution by positive selection in the root branch of the *scn4ab2* clade using this approach. This analysis further supports *scn4ab1* neofunctionalized soon after duplication.

**Fig 3:**
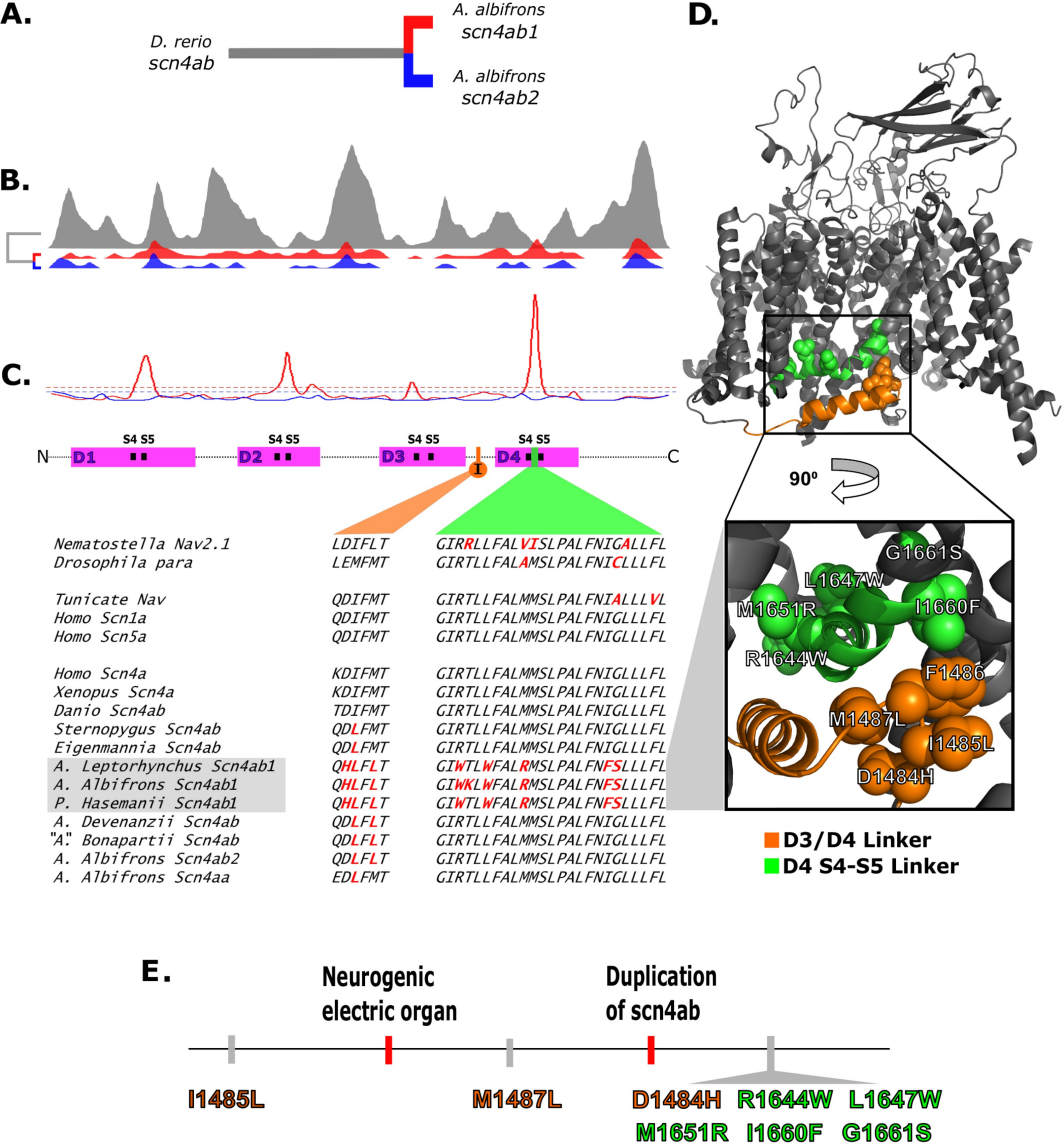
Relative density of amino acid replacements in *scn4ab1* and *scn4ab2*. (A) The orthologs used in the analysis, where the 2 paralogs in *A*. *albifrons* were compared to the much more distantly related ortholog in *D*. *rerio*. (B) Density plots of amino acid substitutions along the sodium channel (N to C) that are specific to each gene. (C) The relative density of substitutions specific to *scn4ab1* and *scn4ab2* (red and blue, respectively). The relative density is the ratio of substitution density at each position in the protein to the added density in the other 2 branches. The dotted lines show conservative significance thresholds for the same relative density measure from simulating random distributions of the amino acid substitutions in the 3 gene sequences. The 4 domains of a typical sodium channel are shown as magenta rectangles, with the transmembrane domains near the highest peak densities indicated in the intracellular linker between S4 and S5 (S4–S5) and the inactivation particle indicated with an orange circle and denoted “I.” The bottom protein alignments are of the inactivation particle (orange) and one of the inactivation receptors in domain 4 (green) that shows the highest relative substitution signal in *scn4ab1*. *Scn4ab1* exhibits several amino acid substitutions that are highly conserved among chordates in both protein structures. Alignments of all human and zebrafish Na_v_ channels are shown in S6 Fig. (D) Structural view of the Na_v_1.4-β1 complex from electric eel with positions of Apteronotid substitution represented as spheres [42]. Residue numbering is from the human cardiac sodium channel, hNa_v_1.5, the background into which mutations were made for electrophysiology in this study. The inset is a magnified view of the putative binding site for the IFM inactivation particle in the D3–D4 loop. (E) Evolutionary sequence of amino acid replacements in the inactivation machinery. C, carboxy-terminus; hNa_v_, human voltage-gated sodium; N, amino-terminus; F, phenylalanine; I, isoleucine; M, methionine.

Na_v_ channels comprise 4 repeating domains (D1–D4), each of which has 6 membrane-spanning helices (S1–S6) (Figs 3A–3C and S6) [34]. The S4 helices in each domain are displaced by membrane depolarization, resulting in a conformation change in the channel that allows an inward flow of Na^+^ ions [34]. Within milliseconds of activation, channel conduction is spontaneously terminated via fast inactivation [34]. The molecular mechanism of this process is unresolved; however, functional experiments have identified several critical molecular features. A hydrophobic triplet of the amino acids isoleucine, phenylalanine, and methionine (IFM) in the intracellular loop between D3 and D4 [35] (the so-called “inactivation particle”) is required for fast inactivation; this motif likely moves in response to membrane depolarization [36], and in the inactivated state, it may interact with a “receptor” containing amino acid side chains in the intracellular S4–S5 linkers of D3 [37] and D4 [38]. Naturally occurring mutations in these regions produce an I_NAP_ that is implicated in neurological and muscular diseases [39–41].

Fig 3 shows the relative density of amino acid substitutions of the Apteronotid *A*. *albifrons scn4ab* paralogs and a distant relative, the *scn4ab* ortholog, in the zebrafish, *D*. *rerio*. Using a minimum mutation parsimony criterion, amino acid changes and indels were mapped onto each of the 3 branches connecting the genes (Fig 3A). For example, if zebrafish *scn4ab* and *A*. *albifrons scn4ab2* have the same amino acid residue at a coding position and *scn4ab1* is different, then an amino acid substitution is assumed to have happened in *scn4ab1* at that position after duplication. With this procedure, the density of substitutions was measured along the length of the 3 proteins (Fig 3B). The relative density of amino acid replacements was measured as the ratio of substitution density in an ortholog to the combined density of replacements of the other 2 proteins at the same position (Fig 3C). Simulations in which substitutions were randomly distributed along sequences were used to generate empirical null distributions of branch-specific substitution densities. A threshold that is reached in <99% of simulations was derived for each protein. Every site in the *scn4ab2* sequence is well below threshold. However, *scn4ab1* greatly exceeds this threshold in 3 locations, each around the S4 and S5 transmembrane regions within domains 1, 2, and 4 (Fig 3C). The strongest signal shows a nearly 10-fold higher density of amino acid substitutions than threshold in the aforementioned D4 S4–S5 linker within the putative inactivation particle receptor. These substitutions accumulated within approximately 2 million years of duplication (Fig 2) despite the strong conservation of these sites across the Na_v_ channel gene family of vertebrates, spanning approximately 550 million years of evolution [43] (S6 Fig).

While this paper was in preparation, a cryo-electron microscopy (CryoEM) structure of the skeletal muscle Na_v_ channel of *E*. *electricus*, the electric eel, was reported [42]. This is the first high-resolution structure of a canonical eukaryotic Na_v_ channel. The state of the channel is not conclusively determined, but it may represent a pre-inactivated conformation wherein the pore is trapped open by an unresolved detergent molecule. This structure suggests extensive interactions between the D3–D4 loop and D4 S4–S5 linker (Fig 3D, green and orange regions). Specifically, the structure predicts intimate association of Apteronotini substitutions in the hydrophobic inactivation particle with those within the D4 S4–S5 linker (Fig 3D inset). Of the substitutions in the D4 S4–S5 linker, 3 are located within a leucine zipper-like motif that has been previously proposed to interact with some part of the D3–D4 loop during fast inactivation [38]. The other two, a highly conserved isoleucine/glycine pair (I1660/G1661, which are F1660/S1661 in the Apteronotini), directly appose the critical phenylalanine (Phe) in the IFM inactivation particle (Fig 3D, inset).

None of the other spinal cord–expressing Na_v_ channels in the Apteronotini (scna8aa, scn8ab, scn1Lab) have amino acid substitutions in these parts of the channel.

### Amino acid substitutions in the channel generate a persistent sodium current

**Fig 4:**
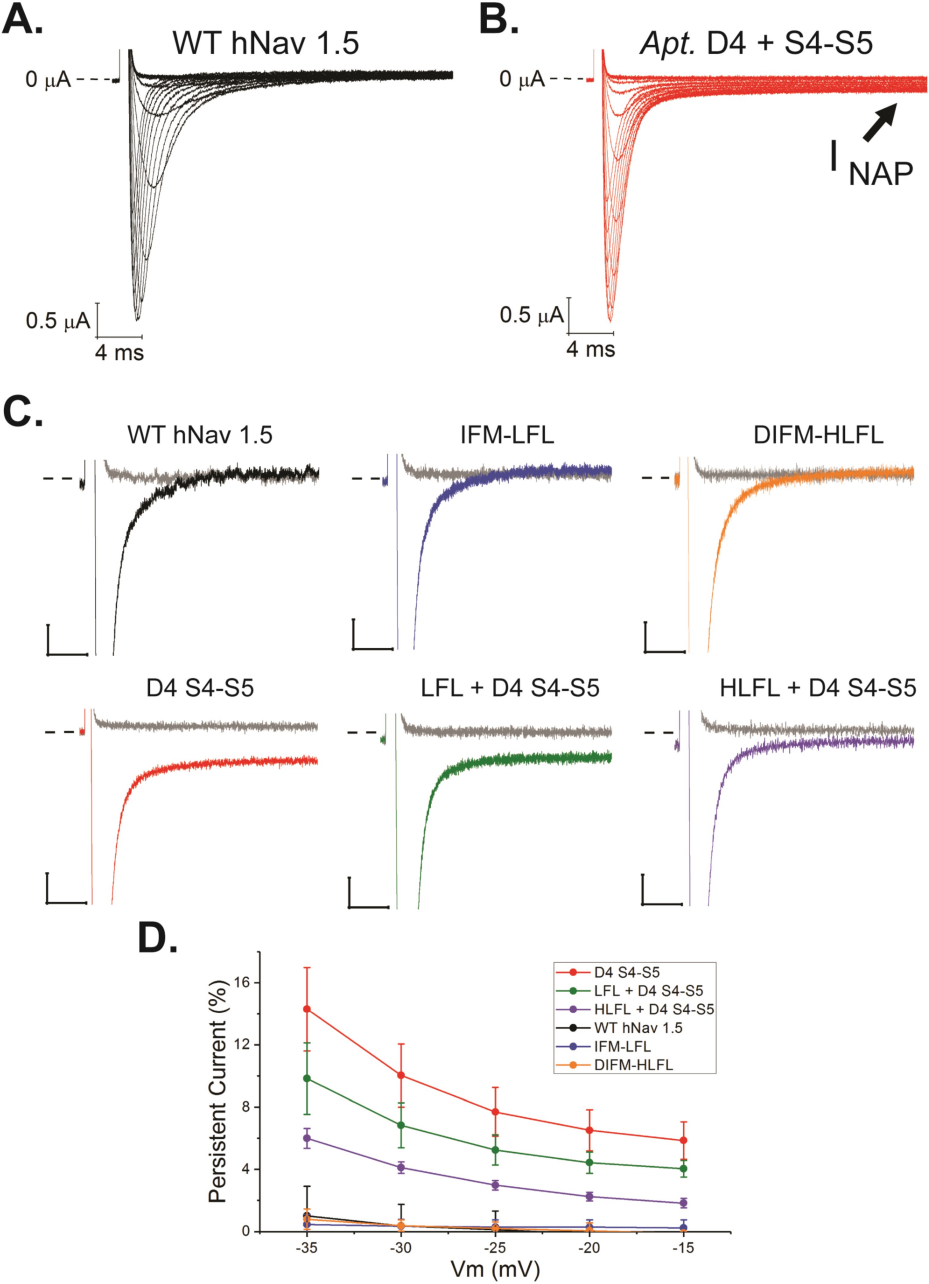
Apteronotini-specific Na_v_1.4b1 (*scn4ab1*) substitutions generate a persistent current in the human cardiac Na_v_1.5. (A) WT hNa_v_1.5 currents elicited by depolarizations in 5 mV steps from −80 mV to +15 mV. (B) Currents generated by an hNa_v_1.5 variant with all 5 Apteronotini-specific substitutions (see Fig 3) in the D4 S4–S5 linker. Note the persistent (non-inactivating) current at the end of the pulse. (C) Normalized exemplar traces of late currents for hNa_v_1.5 mutants. To facilitate comparisons among variants, current responses to steps to −60 mV (gray) and −20 mV (colors) are shown. Scale bars are 5% of peak current (vertical) and 5 milliseconds of time (horizontal), and dashed lines indicate the point of 0 current. (D) Quantification of percentage of persistent current for each variant over a range of membrane potentials. Error bars are ± 1 standard deviation. *N* ≥ 5 for each variant, in S4 Table. For all D4 S4–S5 substitution-containing variants, percentage of persistent current was significantly different (*p* < 0.01) as compared to WT hNa_v_1.5. Figure data included in S2 Data. D4 S4–S5, R1644W/L1647W/M1651R/I1660F/G1661S; DIFM-HLFL, D1484H/I1485L/M1487L; hNa_v_, human voltage-gated sodium; IFM-LFL, I1485L/M1487L; WT, wild-type.

Because the mechanism of inactivation and its structural determinants are highly conserved among animal Na_v_ channels, we made use of an available human Na_v_1.5 sodium channel expression construct, engineered the amino acid substitutions into this channel, and expressed these channels in *Xenopus* oocytes. We predicted that the Apteronotini substitutions would generate an I_NAP_. Wild-type (WT) hNa_v_1.5 channels had no apparent I_NAP_ (Fig 4A). Compared to vertebrates in general, Apteronotid *scn4ab* (protein name; Na_v_1.4b) channels have substitutions in the inactivation particle (IFM → LFL), with that of the Apteronotini *scn4ab1* having 1 further substitution (DIFM → HLFL). None of these substitutions generated an I_NAP_ on their own (Fig 4C, blue and orange traces). The substitution of the complete set of the 5 amino acids observed in the Apteronotini *scn4ab1* channel D4 S4–S5 linker into the WT hNa_v_1.5 linker (Fig 3) caused a large I_NAP_ (Fig 4B). At none of these positions do single alanine mutations generate persistent currents in the neuronal sodium channel Na_v_1.2 [38]. Therefore, the specific chemical nature and/or combination of mutations in this domain of *scn4ab1* are necessary for the physiological persistent current.

The addition of the partial (LFL, found in all Apteronotids) or complete (HLFL, found in Apteronotini) inactivation particle substitutions to the Apteronotini D4 S4–S5 linker substitutions modified the amount of I_NAP_ such that the combination that most resembled the Apteronotid *scn4ab1* channel generated about 6% maximum I_NAP_ (at −35 mV). Importantly, I_NAP_ activated at more hyperpolarized voltages (approximately −60 mV) and was a larger relative percentage of the total current (S7 Fig) in the range from −60 to −40 mV. The channel also showed less steady-state inactivation than the WT hNa_v_1.5 current in this voltage range (S8 Fig). Electrophysiological recordings of spontaneously firing Apteronotid EMNs show that these neurons never completely return to resting potential between action potentials but, instead, briefly reach −40 to −50 mV before the next action potential [19]. This is the voltage range in which I_NAP_ produced by the inactivation apparatus of the Apteronotini *scn4ab1* channels is maximal. Furthermore, the recovery from inactivation, which influences a neuron’s maximum firing frequency, is faster in hNa_v_1.5 (HLFL + D4 S4–S5) than the WT hNa_v_1.5, but only at the earliest (submillisecond) recovery interval (S9 Fig).

## Discussion

Just as the diversification of voltage-gated ion channels contributed to diversification of animal nervous systems, the evolution of novelty in the Na_v_ channel gene family led to diversification of electric organ signals [24,26,44]. In both major clades of electric fish (Gymnotiformes, Mormyroidea), a muscle-specific Na_v_ channel gene (*scn4a*) that duplicated in the whole-genome duplication at the origin of teleosts retained muscle expression for approximately 100 million years before it was convergently recruited into the electric organ at the origin of both groups of electric fish. A clade of electric fish within the South American Gymnotiformes exhibits another more recent duplication at one of the muscle-expressing Na_v_ channel loci; this time, *scn4ab* duplicated to *scn4ab1* and *scn4ab2*. This lineage of electric fish is specialized for high-frequency discharges from a unique electric organ composed of the axons of EMNs. Shortly after duplication, *scn4ab1* gained expression in the spinal cord, where the EMN electric organ is located, and it shows comparative sequence patterns consistent with having evolved under positive selection (Fig 2).

The EMNs of the neurogenic electric organ of some Apteronotids, including those that contain *scn4ab1*, fire spontaneously and at the fastest rate known in any animal neuron. Our molecular evolution (Fig 3) and biophysical (Fig 4) analyses suggest that several amino acid substitutions within the inactivation particle and particle receptor lead to the generation of I_NAP_ in *scn4ab1*. The magnitude of I_NAP_ we measured in hNa_v_1.5—that was specifically attributable to the Apteronotini substitutions—was similar in magnitude to the I_NAP_ observed in spontaneously firing mammalian Purkinje neurons, wherein I_NAP_ equal to only a small percentage of the peak current [45,46] is sufficient to drive spontaneous firing [47,48]. Because pharmacological and electrophysiological data from Apteronotus EMNs indicate the presence of a persistent sodium current, this strongly supports the origination of *scn4ab1* as an important molecular contributor to the evolution of EMN spontaneous high-frequency electric signals in the Apteronotini [3]. It is, however, important to note that the I_NAP_ measured in spontaneously firing Purkinje neurons is elicited by the interaction of the pore-forming Na_v_ subunit with an auxiliary subunit protein [49]. We propose that the Apteronotid EMNs use a distinct mechanism, wherein substitutions within *scn4ab1* are sufficient to generate I_NAP_.

The inactivation particle and receptor evolved on different evolutionary timescales (Fig 3E). The substitutions observed within the putative *scn4ab1* inactivation receptor (D4 S4–S5 linker) happened soon after the duplication of *scn4ab*; when substituted into the human Na_v_1.5 channel, they cause a large I_NAP_ (Fig 4C left). The inactivation particle, however, evolved over a longer timescale, with 2 of the 3 substitutions preceding the duplication. One of the amino acid substitutions (IFM to LFM) occurred before the evolution of Gymnotiformes. The basal Apteronotid then gained another substitution (LFM to LFL). When substituted alone into human Na_v_1.5, these substitutions have no discernable impact on inactivation. When coupled with the 5 mutations in the putative receptor, however, they have a moderate mitigation of the persistent current (Fig 4C middle). This suggests that the initial substitutions in the gating particle may have been benign to start but later interacted with and possibly facilitated substitutions in the receptor when *scn4ab* duplicated. One more substitution found near the canonical inactivation particle, DLFL to HLFL, occurred around the same time as the mutations found in the receptor. The addition of the HLFL-to-DLFL substitution further mitigates the persistent current (Fig 4C right). The central F in the IFM particle remains conserved, which is consistent with data indicating that it has a much larger impact on inactivation than its flanking amino acids [35].

The impact of mutations from different evolutionary time periods suggests that *scn4ab1* arose and played a role in the electric organ rather than a spinal cord Na_v_ channel because its inactivation particle had acquired mutations that dampened the impact of receptor mutations on the electric organ. Despite the extensive changes in the 2 interacting domains, our experiments indicate that the functional interaction between inactivation particle and receptor is maintained in *scn4ab1*, suggesting great evolutionary lability post duplication in these usually highly conserved parts of the channel.

The gain of expression of *scn4ab1* in the spinal cord may have caused or was caused by the changes in the inactivation gate specific to the Apteronotini. Alternatively, the gain in expression might have occurred because of the duplication itself. Genomic and transcriptomic investigations into more basal Apteronotini species will illuminate the coevolution of expression and the inactivation machinery in this gene.

One puzzling observation in our data is that, despite *scn4ab1* displaying an I_NAP_, this gene still maintains abundant expression in the skeletal muscle, where presumably a persistent current would be disruptive to swimming behavior. Mutations in the *scn4a* gene in humans that results in I_NAP_ in muscles cause muscle diseases [50]. A critical question is this: what prevents an I_NAP_ and spontaneous firing in muscle? There are many posttranscriptional mechanisms for compensating for this expression. I_NAP_ may be inhibited by other muscle-expressing Na_v_ channel–associated proteins, known as beta subunits [3,4] (which differ in their expression in the Apteronotid muscle and spinal cord [S10 Fig]). Translation of *scn4ab1* may be suppressed by muscle-specific micro-RNAs (miRNAs), or there may exist compensatory increases in hyperpolarizing ionic currents that are specific to the Apteronotini. Finally, it is possible that there is an I_NAP_ in Apteronotini muscle and that the current is part of the unique swimming patterns exhibited by these fish. Most electric fish species, and especially those that maintain high-frequency signaling, remain rigid during swimming and rely on the undulation of a long, derived anal fin. Perhaps a persistent current in these fishes’ muscle contributes to their muscle rigidity. These explanations are highly speculative at this time but may inspire further study.

While the duplication of an scn4ab gene and its evolution likely underlies the ability of the Apteronotini to spontaneously fire their electric organs at high frequencies, some other Apteronotid species generate high-frequency electric organ signals often exceeding 1,000 Hz, although whether they fire spontaneously is unknown. It is also possible that another spinal cord–expressing channel (e.g., *scn8ab*) has evolved the ability to support high-frequency firing in other species. Exploration of the sodium channel gene families of other Apteronotids will likely be a rich vein for future research. A complete understanding of EMN firing rates in the Apteronotini must also consider the activities of the other Na_v_ channels expressed in EMNs, such as *scn8aa* and *scn8ab*, as well as the voltage-gated potassium channels expressed in these neurons. It will be interesting to know whether any of these other channels have also evolved mechanisms for rapid spontaneous firing and/or whether there are other examples of “cryptic duplication” in these channels. Identifying such highly similar paralogs as *scn4ab1* and *scn4ab2* requires a level of granularity in transcriptomic analysis that has historically been difficult to achieve.

In this study, we have presented evidence that the continued divergence and diversification of electric organ signals is driven in part by the repeated duplication and neofunctionalization of a Na_v_ channel gene. In 3 separate fish lineages, a duplicate originating from a muscle-type sodium channel was co-opted by a novel electric organ, twice by a muscle-derived organ and once by a neurogenic organ. It is surprising to see *scn4a*-type sodium channels involved in neurogenic electric organs rather than one of the sodium channel types normally expressed in spinal neurons, namely *scn8ab*. The recurring neofunctionalization of *scn4a* genes suggests that this type of sodium channel may be relatively free of selective constraints to evolve novel expression and structural innovations [22]. Phylogenetic placement of the many mutations involved in *scn4ab1* inactivation elicits an interesting hypothesis that perhaps some preadaptive mutations in the inactivation machinery primed this channel for evolution in Apteronotid EMNs.

Perhaps the genetic substrate for new adaptive phenotypes will be found in gene types and families not necessarily expressed in the tissues from which novel phenotypes are derived but more from types of genes whose duplication and neofunctionalization are less detrimental to the organism. Further research may find that the scn4a-type sodium channel displays this remarkable tendency to contribute to electric fish evolution because, compared to other sodium channels, it is the least disruptive to the organism when it duplicates and neofunctionalizes.

## Methods

### Sample preparation

Animals were acquired through the aquarium trade. Fish were euthanized according to ethical guidelines set by IACUC at UT Austin and Indiana University Bloomington. Skeletal muscle samples were taken from the midtrunk hypaxial location and the spinal cord from the mid to tail location of 3 adult *A*. *albifrons*, *I*. *punctatus*, and *E*. *virescens*, as well as 1 adult *A*. *leptorhynchus* and *P*. *hasemanii*. Tissue samples were flash frozen in liquid nitrogen, and total RNA was extracted and DNA removed, following previously described protocols [23]. Total RNA samples were submitted to the University of Texas at Austin core genomics facility, where ribosomal RNA was removed; paired-end 100 bp RNA-seq (*A*. *albifrons* and *I*. *punctatus*) or paired-end 150 bp RNA-seq (*E*. *virescens*, *A*. *leptrohynchus*, and *P*. *hasemanii*) was performed on an Illumina HiSeq 2000 to produce between 30 and 34 million reads per sample.

### De novo transcriptome assembly and sodium channel family annotation

Raw reads were quality filtered and adapter trimmed with Trimmomatic v 0.35. Muscle and spinal cord reads were combined and transcripts assembled from a single biological replicate from each species using Trinity [51] v. 2.0.6. Alpha and beta sodium channel transfrags were extracted and annotated using reciprocal blast. All 8 sodium channel alpha subunits and 5 beta subunits from the *D*. *rerio* were downloaded from GenBank and used to blast sodium channel sequence from each of the transcriptomes with e-value threshold e-6. This yielded 38–71 (alpha) and 17–45 (beta) transfrags per species. CDS’s of transfrags were found using Transdecoder v. 3.0.0 with minimum CDS of 100 amino acids. CD-hit-est v. 4.6.4 was used to cluster all coding sequences with >99% sequence similarity and >90% of the sequence overlaps with the longest sequence in each cluster. This resulted in 12–25 transfrags for the alpha subunits and 7–9 for the beta subunits in each species. These sequences were then blasted against the *D*. *rerio* proteome (assembly GRCz10) with blastx (BLAST+ V 2.2.28) where the top hit was designated the transfrag’s ortholog.

### *Scn4ab1* and *scn4ab2* assembly and annotation

The quality of transfrags was visually inspected by mapping reads (RSEM; see below) and using Integrative Genome Browser v 1.3.1 to inspect patterns among mapped reads. The quality was generally good, except for the scn4ab transfrags in the Apteronotid species. Reads from muscle showed high-frequency base mismatches distributed uniformly along the length of the transfrags. Inspection of the reads showed that all high-frequency mismatched bases existed within the same reads. This same pattern occurred in all 3 Apteronotids investigated. However, in all 3 species, these “polymorphisms” did not appear in mapped reads from the spinal cord samples, suggesting the presence of a duplicate of *scn4ab* that did not assemble well and is only expressed in muscle. The facts that more than half of the gene sequence was assembled in each species and that there were very few polymorphisms of reads mapping in the spinal cord together indicated that, while one paralog did not assemble very well, the other did and contained little or no chimeric sequence with its paralog. There were small fragments (<1 kb) that corresponded to the second duplicate in some of the Apteronotid species. To better assemble this gene, we extracted all reads that mapped to *scn4ab* in muscle with at least 2 mismatches and used SOAPdenovo-Trans [52] with kmer size of 55 to assemble large transfrags of this gene. After adding this gene to the transcriptome and remapping with RSEM, the polymorphisms largely disappeared from scn4ab in skeletal muscle in each species. The presence of duplicate scn4ab genes was confirmed with PCR and Sanger sequencing of DNA samples from *A*. *albifrons*.

### Expression analysis

All sodium channel transfrags were extracted from the transcriptome and analyzed with RSEM [53] v. 1.2.28 to estimate the relative expression in transcripts per million (TPM) for each transfrag. To estimate gene-level expression, alternative splice forms as estimated by Trinity (_i notation in Trinity V. 1.2.28 notation) were summed for each gene (_g in Trinity notation). When multiple loci (_g) were annotated as orthologs of the same gene, they were assumed to be fragments of the same locus at different, mostly nonoverlapping locations in the gene. Expression levels were alternatively estimated as the averaged TPM or maximum TPM across all these transfrags for each gene in each replicate. These 2 approaches yielded very similar relative expression patterns to the averaging approach just described (S2 Fig). *E*. *electricus* expression was in units of FPKM. To make relative expression comparable between species, TPM or FPKM values were scaled by total sodium channel expression in each tissue.

### qPCR analysis

Three muscle and spinal cord total RNA samples from *A*. *albifrons* were reverse transcribed to cDNA with random hexamers and oligo-dT20 primers using the superscript III kit and standard protocol (Life Technologies, Grand Island, NY). Primers and hydrolysis probes (IDT, Coralville, IA) specific to *scn4ab1*, *scn4aa*, and the housekeeping gene *RPL13a* (which was sequenced using degenerate primers, PCR, and Sanger sequencing) were used with the TaqMan Universal Master Mix NO UNG (Applied Biosystems, Branchburg, NJ) to perform qPCR reactions in the 2 tissues of 3 biological replicates. Specificity of primers and probes was confirmed through PCR and Sanger sequencing. qPCR amplicons were designed to span multiple exons, and negative controls were performed where the reverse transcriptase was not added to the reaction mix. qPCR reactions were run on a Viia7 Real-Time PCR machine (Applied Biosystems).

*RPL13a* normalized expression levels were estimated for *scn4aa* and *scn4ab1* in muscle and spinal cord using previously published protocol [23]. In brief, the raw amplification data were baseline corrected, and linear regression on the log-linear phase of amplification for each individual reaction was used to verify close to 100% doubling efficiency and select a common threshold for each sodium channel gene and the housekeeping gene. Cq and doubling efficiency was estimated and confirmed using the LinRegPCR software package [54–56]. Normalized expression was estimated using 2^-ΔΔCq^ method [57], which assumes 100% doubling efficiency.

### Phylogenetic analysis

*Scn4ab* sequences were trimmed to their longest coding sequence with stop codons removed for codon alignment. A codon alignment was created on the GUIDANCE2 server. A GUIDANCE alignment quality threshold of 0.93 was selected, which removed 20% of the codon positions. We used MRBayes version 3.2.5 [58] to estimate the gene tree topology. We estimated the gene tree by model averaging over the space of all possible GTR models with gamma-distributed rates and data partitioned by the 3 codon positions (lset nst = mixed rates = gamma; mcmc ngen = 1,000,000). This analysis produced a consensus tree that was identical in topology and nearly identical in branch lengths to a tree generated without partitioning the codon positions. The consensus tree was very well resolved. All branches had posterior probability >0.99 except one, which had posterior probability of 0.75. Two independent runs, each with 4 chains, converged for 2 million iterations after 1 million burn-in on identical marginal posterior distributions for all model parameters. Posterior distributions were found to be of good quality when visualized with Tracer. Maximum likelihood estimates of branch dN/dS were estimated for the majority rule consensus tree generated from unpartitioned codon alignment using the BSR method [32] on the Datamonkey server. Sequences used in this analysis are given in S1 Table. Maximum likelihood dN/dS was also estimated using PAML [33] version 4.9e with F3X4 for codon frequencies. The following two different models were evaluated: (1) all branches share the same dN/dS ratio and (2) the root branch of the *scn4ab1* clade having a unique ratio. Models were compared by the likelihood ratio test.

### Amino acid substitution density analysis

To locate regions where the duplicate sodium channels may have diverged in function, the density of amino acid substitutions along the peptide sequence was analyzed. The amino acid sequences of *scn4ab1* and *scn4ab2* were most complete in *A*. *albifrons*. These sequences were aligned against *scn4ab* of *D*. *rerio*. Substitutions or indels were mapped to each of the 3 branches of the resultant tree by a minimum mutation parsimony criterion. At positions where all 3 sequences differed, a substitution was assumed to have occurred on each of the 3 branches. This slightly inflates the density of substitutions at these regions for the whole tree but does not impact investigation of the relative density of amino acid substitutions. For each of the branches connecting the 3 genes to the internal node, the density of substitutions at each residue was calculated using the R function *density* (package *stats*) with binwidth set to 15 and then weighted by that gene’s share of the total number of substitutions inferred by parsimony. The relative density at position x on sequence y was calculated as the density of position x on sequence y divided by the sum of the density of the other 2 sequences at the same position. For example, if *scn4ab1* has a relative density of 2 at a position in the sequence, then the density of amino acid substitutions is twice that of the other 2 sequences combined. We generated 10,000 simulations in which the number of amino acid substitutions mapped to each branch were randomly distributed along each of the 3 sequences, and the same relative density statistic was measured to get a NULL distribution for relative density and to determine if the clustering pattern of amino acid substitutions observed is unlikely to have arisen by chance.

### Molecular clock analysis

The dataset included sequences from 37 taxa for 3 genes: cytochrome c oxidase subunit I (COI), cytochrome B (CytB), and recombination activating gene 2 (RAG2). Most sequences were derived from concatenated consensus data (see S2 Table of the manuscript for a full list of individuals, source IDs, and accession numbers), with supplemental Apteronotid sequences coming from [31] and outgroup sequences taken from NCBI (accession numbers for supplemental Apteronotid and outgroup sequences listed in S2–S3 Tables). Sequences were concatenated and aligned using the L-INS-I protocol in MAFFT [59]. Maximum likelihood trees were estimated using a GTR + gamma substitution model in RAxML v. 7.4.2 [60] with *D*. *rerio* as the outgroup. Dating estimates were performed using the RelTime method [61] and a Tamura-Nei model [62] in the MEGA7 software package [63]. The model included 5 discrete gamma estimates, allowed for invariant sites, included all codon positions, and discarded alignment gaps. The tree was calibrated using 2 dating ranges estimated from [64]: (i) the split between *I*. *punctatus* and *Serrasalmus maculatus* was assigned a potential range of 115–150 MYA, and (ii) the split between *S*. *maculatus* and *Orthosternarus tamandua* was assigned a potential range of 75–115 MYA.

### Electrophysiology

Two electrode voltage clamp recordings in *Xenopus* oocytes were performed at 20–22°C using a Turbotec 03X amplifier (NPI). Intracellular recording electrodes had resistances of 0.2–0.4 MΩ when backfilled with 3 M KCl. Voltage protocols are described in detail in the figure legends. For all experiments, the holding potential was −120 mV. Mutations were made into human cardiac sodium channel Na_v_1.5 in pcDNA 3.1 using mini-gene synthesis (Biobasic, Markham, Ontario, Canada) in conjunction with Gibson Assembly. All constructs were verified by sequencing through the entire open reading frame. RNA was transcribed using the Mmessage Mmachine T7 Ultra Kit (Thermofisher, Waltham, MA) after linearization with Not1. Approximately 12.5–50 ng of cRNA from the alpha subunit was co-injected with 6.25–25 ng of cRNA from the β1 auxillary subunit, which is expressed ubiquitously in excitable cells and enhances sodium channel surface expression [65,66]. Recordings were conducted 24 to 48 hours later in oocyte Ringer’s solution containing (in mM): 116 NaCl, 2 KCl, 1.8 CaCl_2_, 2 MgCl_2_, 5 mM HEPES, pH 7.4. Traces were acquired at 50 Khz and filtered at 10 Khz for display in the figures. For calculation of persistent current, pClamp 9.2 was used to average the last 0.5 ms of each 30-ms pulse, and then this value was divided by the peak of the transient component, after linear leak subtraction. All statistical comparisons were by unpaired Student *t* test with two-tailed distribution.

## Supporting information

S1 Fig The axons of the brainstem pacemaker neurons run down the spinal cord (grey line) and synapse on the EMNs (teal). Top: in most adult Gymnotiforms, the axons of the EMNs synapse on the muscle-derived cells of the electric organ (salmon). In Apteronotids, the muscle-derived electric organ degenerates (former position faded), and the axons of the EMNs extend and form a new, neurogenic electric organ. EMN, electromotorneuron.
(TIF)

S2 Fig The relative expression of sodium channels in muscle and spinal cord. Plots show the relative expression of all transfrags of each paralog in each species. For most paralogs, each transfrag has similar expression. The unduplicated *scn4ab* ortholog in non-Apteronotids is highlighted in purple, while the duplicate *scn4ab* parlaogs in Apteronotids are highlighted in red and blue. Figure data included in S1 Data.
(TIF)

S3 Fig Relative expression of *scn4aa* and *scn4ab1* in muscle and spinal cord derived from qRT-PCR in samples from *A*. *albifron*s. qRT-PCR, quantitative reverse transcription PCR. Expression was normalized to the housekeeping gene RPL13a.
(TIF)

S4 Fig Sequence alignment of *A*. *albifrons* muscle-specific *scn4ab2* (top) and EMN/muscle–expressing *scn4ab1* (bottom). Sequences derived from molecular cloning and Sanger sequencing. Dashed vertical lines indicate exon–intron boundaries. Exon numbering is according to alignment with *D*. *rerio scn4ab*. EMN, electromotorneuron.
(TIF)

S5 Fig Gene tree for *scn4ab* of Gymnotiforms and other teleosts rooted with scn4aa. Note that scn4ab is in single copy in 2 basal Apteronotids (*“A” bonapartii* and *A*. *devenanzi*) but duplicated into *scn4ab1* and *scn4ab2* before the divergence of the 3 members of the Apteronotini. Key events in the evolution of myogenic and neurogenic electric organs are noted. Posterior probabilities given for duplication of *scn4ab* in the Apteronotini. Gene accessions included in S1 Table.
(TIF)

S6 Fig The domain 3–4 intracellular loop and the domain 4 S4–S5 linker from Na_v_ channels of *A*. *albifrons*, *D*. *rerio*, and *Homo sapiens* to illustrate the strong conservation of these motifs across channels and species. Some amino acid substitutions occur in all Apteronotid *scn4ab* channels (blue dot), some in all Apteronotini (red dots), and 1 only in *A*. *albifrons* (green dot). Na_v_, voltage-gated sodium.
(TIF)

S7 Fig Normalized current–voltage relationship for the transient and persistent current for the cell recorded in Fig 4B. Note that the persistent current activates at a more negative voltage than the peak current and that it comprises a relatively greater fraction of the peak current in the range from −60 to −40 mV. Figure data included in S2 Data.
(TIF)

S8 Fig Effects of Apteronotinti Na_v_1.4ab2 substitutions on equilibrium gating of human cardiac Na_v_1.5. A. Inactivation particle substitutions (see Figs 3 and S6) did not affect channel activation. (B) Apteronitini-specific domain 4 S4–S5 substitutions were associated with a 7–10 mV depolarizing shift in channel activation either alone or in conjunction with substitutions in the inactivation particle. (C) Effects of Apteronotini (DIFM to HLFL) inactivation particle substitutions on steady-state inactivation. *Xenopus* oocytes expressing sodium channel variants were subjected to a 500-millisecond conditioning pulse at a given voltage, followed by a 1-millisecond step at −100 mV and a 20-millisecond test pulse at −20 mV. (D) Same as panel C but showing effects of Apteronotini domain 4 S4–S5 substitutions on steady-state inactivation alone and in conjunction with substitutions in the inactivation particle. Note the nonzero asymptotes for steady-state inactivation in the D4 S4–S5 variants. *N* ≥ 5 for each variant; quantification in S4 Table. Figure data included in S2 Data. D, aspartate; F, phenylalanine; H, histidine; I, isoleucine; M, methionine; Na_v_, voltage-gated sodium.
(TIF)

S9 Fig Apteronotinti Na_v_1.4ab substitutions in domain 4 S4–S5 sped recovery from fast inactivation of hNa_v_1.5, which is partially mitigated by substitutions in the inactivation particle. Sodium channel currents were activated via 20-millisecond pulses to −20 mV, which were separated by a recovery interval at −120 mV for a specified length of time. (A) Normalized fraction of current as a function of recovery interval for WT hNa_v_1.5 and Apteronotinti inactivation particle substitutions. (B) Same as panel A but showing effects of Apteronotinti Na_v_1.4ab domain 4 S4–S5 substitutions on recovery from fast inactivation alone and in conjunction with knifefish substitutions in the inactivation particle. (C) Quantification of fraction of current recovered at the shortest recovery interval (0.5 milliseconds). Asterisk indicates variants with statistically significant differences (*p* < 0.01) as compared to WT hNa_v_1.5. (D) Same as panel C but showing fraction recovered by 1.0 milliseconds. *N* ≥ 4 for each variant. In panel C and D, note that the domain 4 S4–S5 substitutions (red bar) increased the fraction of current recovered as compared to hNa_v_1.5 (indicating faster recovery from inactivation). Inclusion of the inactivation particle substitutions (green and purple bars) reduced this effect. Figure data included in S2 Data. hNa_v_, human voltage-gated sodium; WT, wild-type.
(TIF)

S10 Fig Beta subunits of muscle and spinal cord. Beta subunits are known to modify the properties of Na_v_ channels. Note that different subunits are expressed in the muscle and spinal cord of Apteronotids. Figure data included in S1 Data. Na_v_, voltage-gated sodium.
(TIF)

S1 Table Gene accessions for gene tree and expression analysis. (DOCX)

S2 Table Accession for genes used for chronogram. (DOCX)

S3 Table Supplemental sequences for chronogram. (DOCX)

S4 Table Activation and steady-state inactivation statistics. (DOCX)

S1 Data Data from expression and phylogeny figures. (XLSX)

S2 Data Data from electrophysiology figures. (XLSX)

## Abbreviations

BSR: Branch-sites REL
C: carboxy-terminus
COI: cytochrome c oxidase subunit I
CryoEM: cryo-electron microscopy
CytB: cytochrome B
D: aspartate
dN/dS: nonsynonymous replacements per nonsynonymous site divided by the synonymous changes per synonymous site
EMN: electromotorneuron
EO: electric organ
F: phenylalanine
H: histidine
hNa_v_: human voltage-gated sodium
I: isoleucine
I_NAP_: persistent sodium current
L: leucine
M: methionine
miRNA: micro-RNA
MYA: million years ago
N: amino-terminus
Na_v_: voltage-gated sodium
PAML: phylogenetic analysis by maximum likelihood
Phe: phenylalanine
qPCR: quantitative PCR
RAG2: recombination activating gene 2
REL: random effects likelihood
TPM: transcripts per million
WT: wild-type
