# Supplementary figures and images for: Rapid evolution of a voltage-gated sodium channel gene in a lineage of electric fish leads to a persistent sodium current
Source: PLoS Biol. 2018 Mar 27;16(3):e2004892. doi: 10.1371/journal.pbio.2004892 (PMC5870949; doi:10.1371/journal.pbio.2004892)

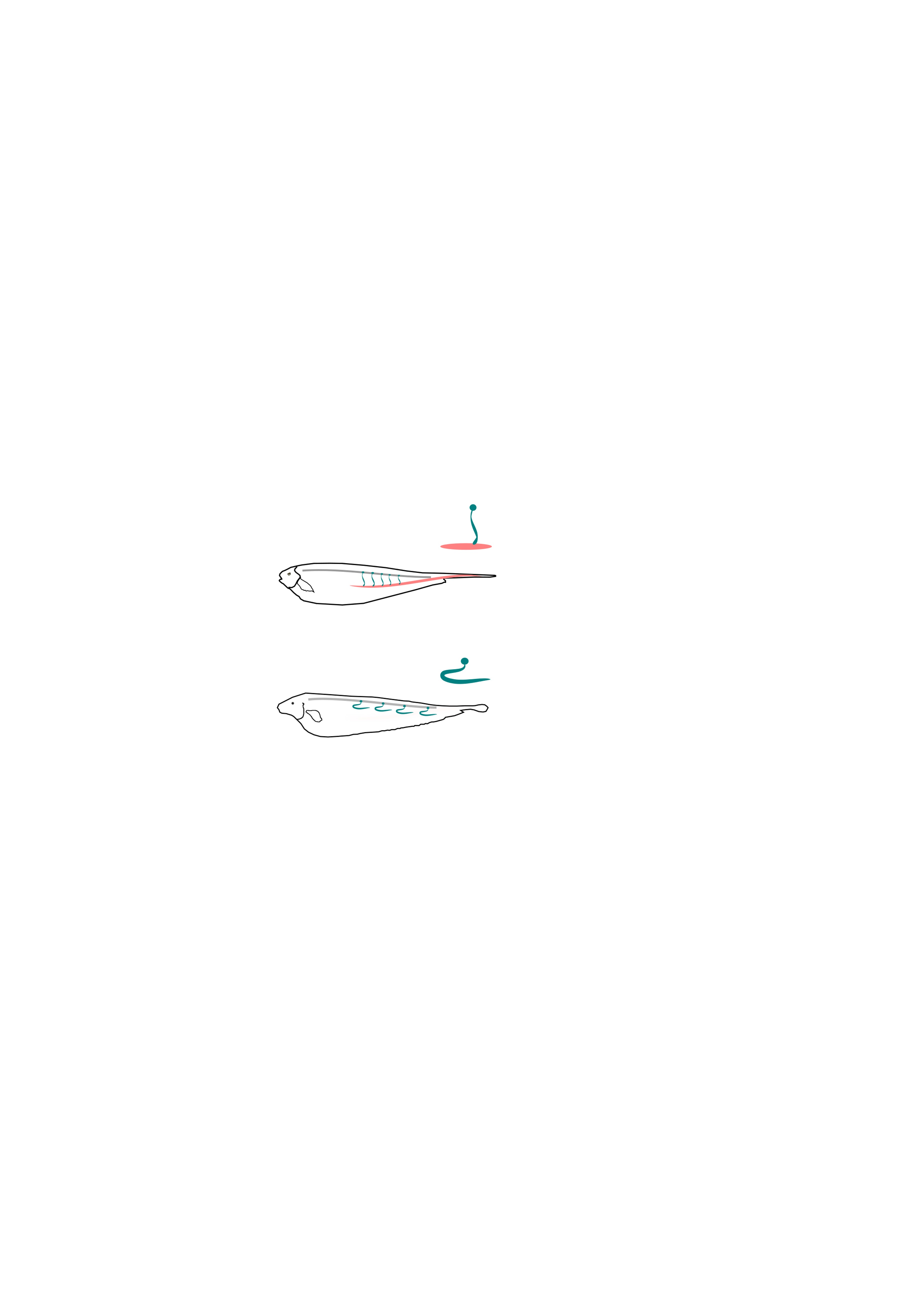

Supplement: S1 Fig — Top: in most adult Gymnotiforms, the axons of the EMNs synapse on the muscle-derived cells of the electric organ (salmon). In Apteronotids, the muscle-derived electric organ degenerates (former position faded), and the axons of the EMNs extend and form a new, neurogenic electric organ. EMN, electromotorneuron. (TIF) [file pbio.2004892.s001.tif]

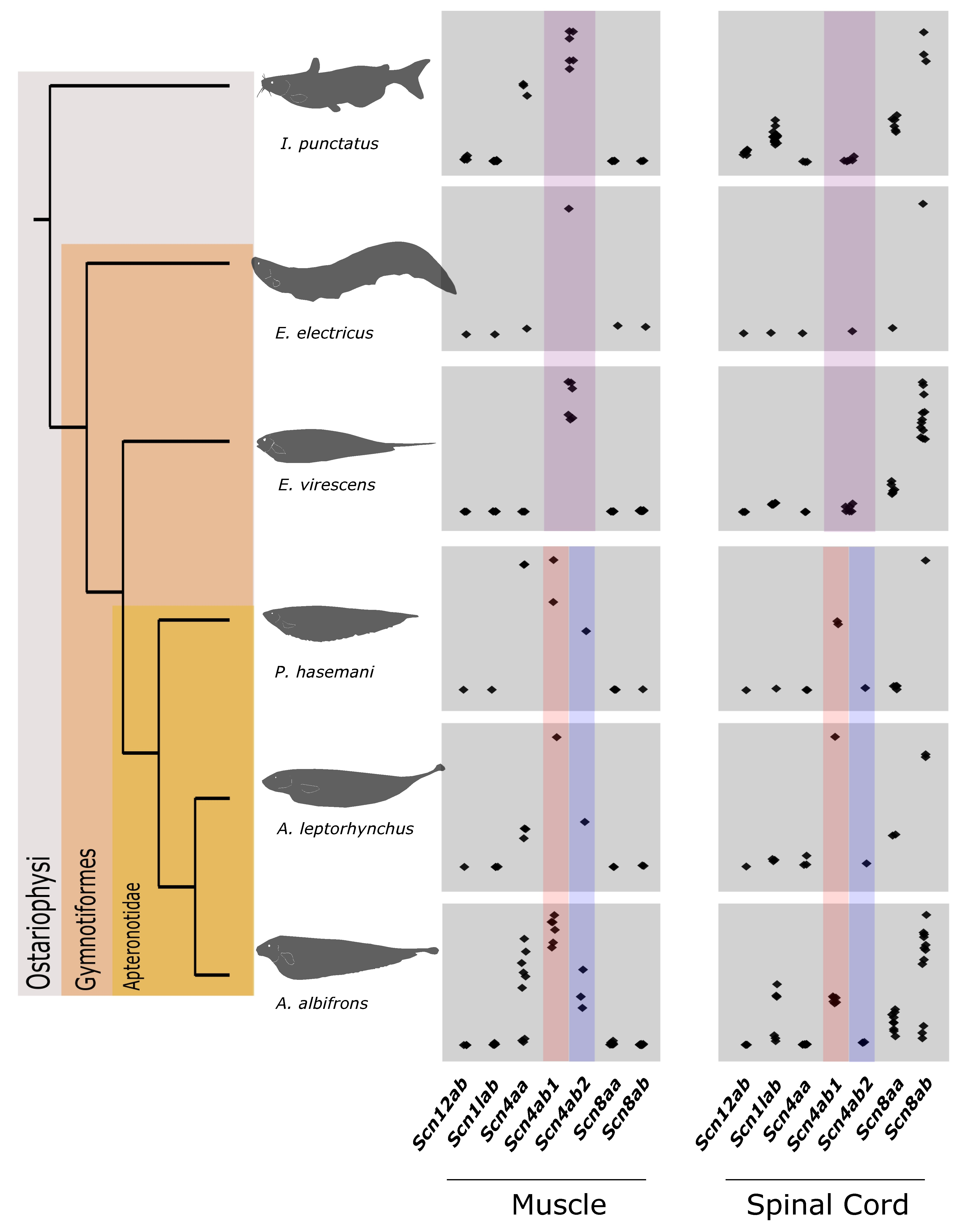

Supplement: S2 Fig — Plots show the relative expression of all transfrags of each paralog in each species. For most paralogs, each transfrag has similar expression. The unduplicated scn4ab ortholog in non-Apteronotids is highlighted in purple, while the duplicate scn4ab parlaogs in Apteronotids are highlighted in red and blue. Figure data included in S1 Data. (TIF) [file pbio.2004892.s002.tif]

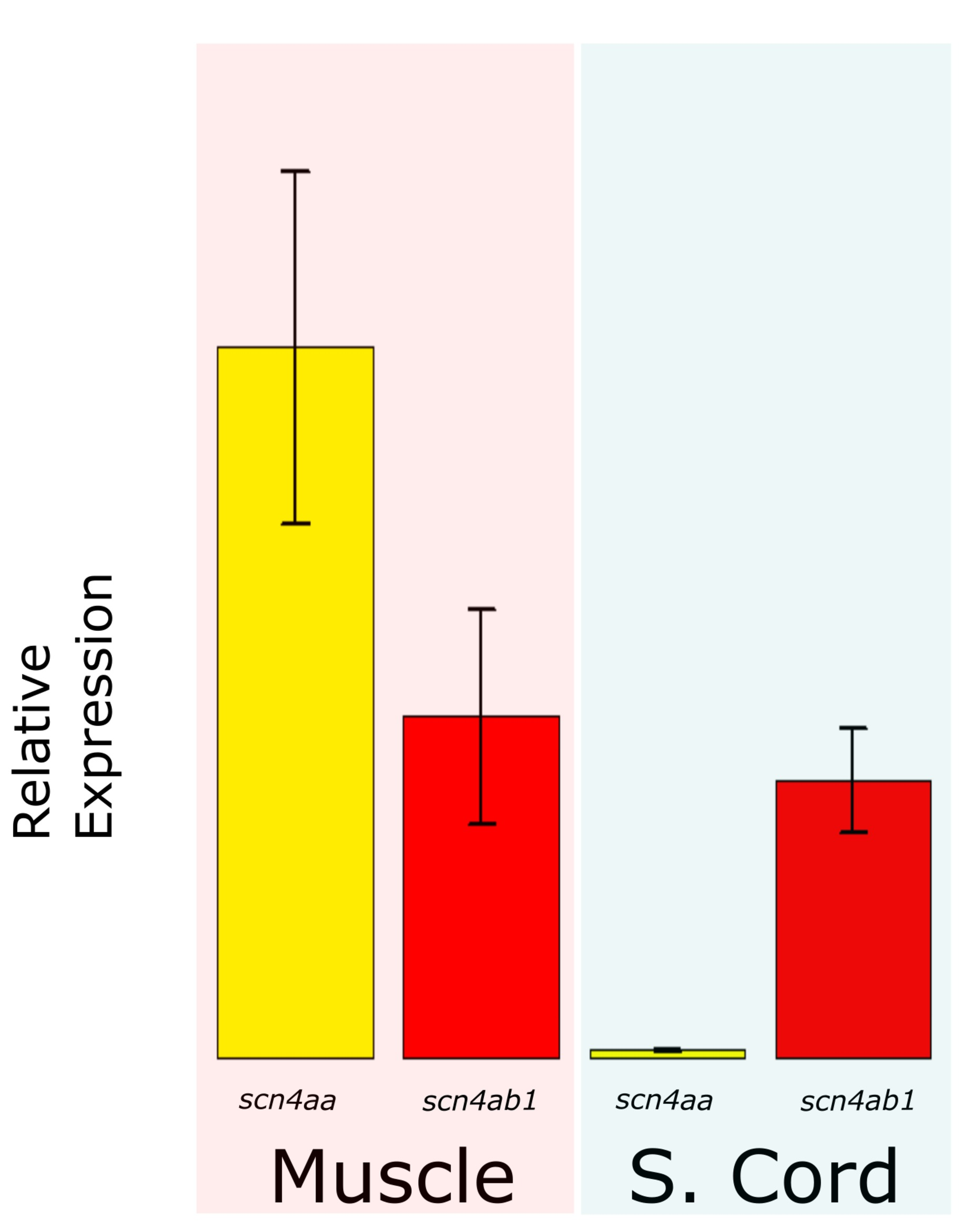

Supplement: S3 Fig — qRT-PCR, quantitative reverse transcription PCR. Expression was normalized to the housekeeping gene RPL13a. (TIF) [file pbio.2004892.s003.tif]

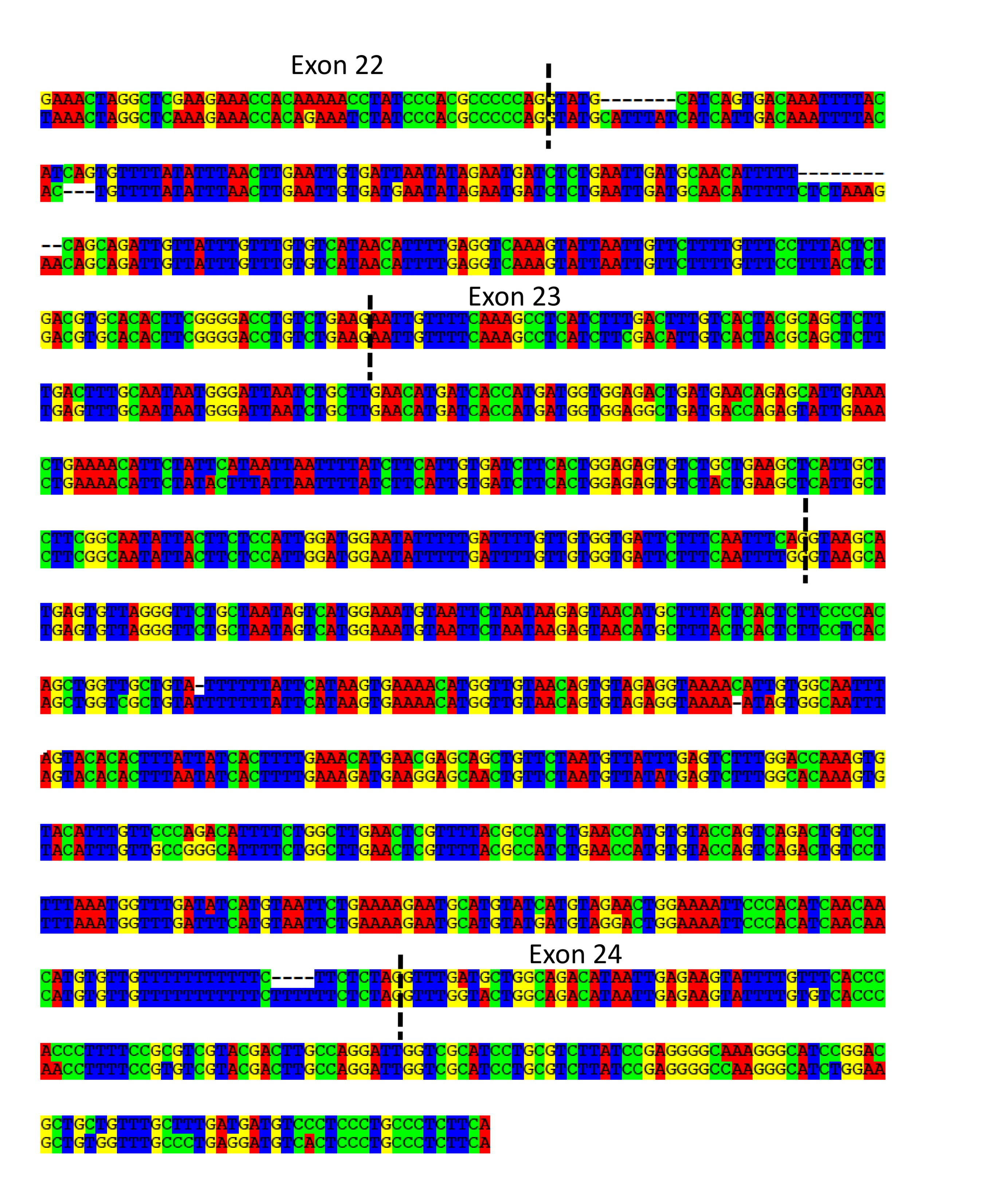

Supplement: S4 Fig — Sequences derived from molecular cloning and Sanger sequencing. Dashed vertical lines indicate exon–intron boundaries. Exon numbering is according to alignment with D. rerio scn4ab. EMN, electromotorneuron. (TIF) [file pbio.2004892.s004.tif]

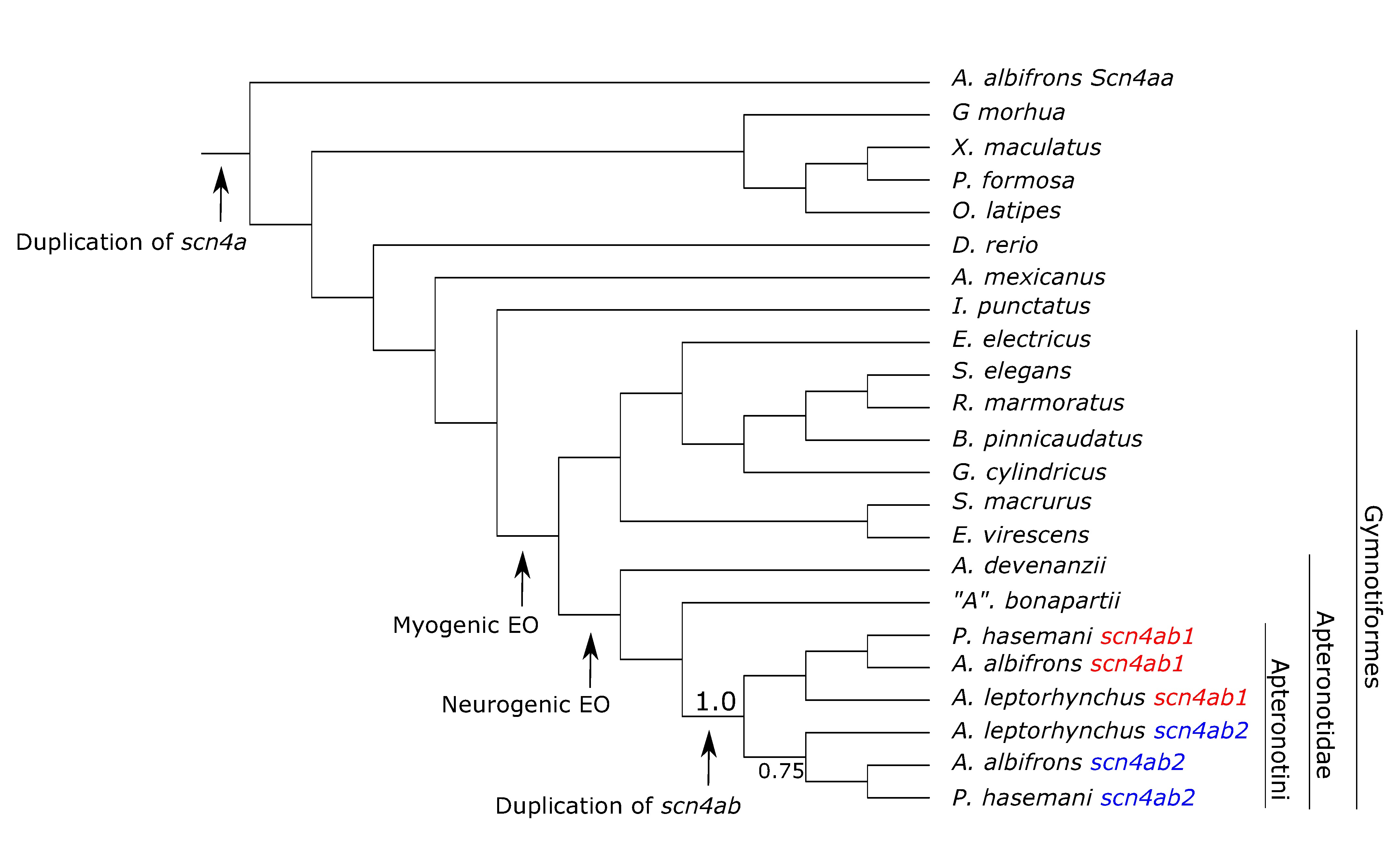

Supplement: S5 Fig — Note that scn4ab is in single copy in 2 basal Apteronotids (“A” bonapartii and A. devenanzi) but duplicated into scn4ab1 and scn4ab2 before the divergence of the 3 members of the Apteronotini. Key events in the evolution of myogenic and neurogenic electric organs are noted. Posterior probabilities given for duplication of scn4ab in the Apteronotini. Gene accessions included in S1 Table. (TIF) [file pbio.2004892.s005.tif]

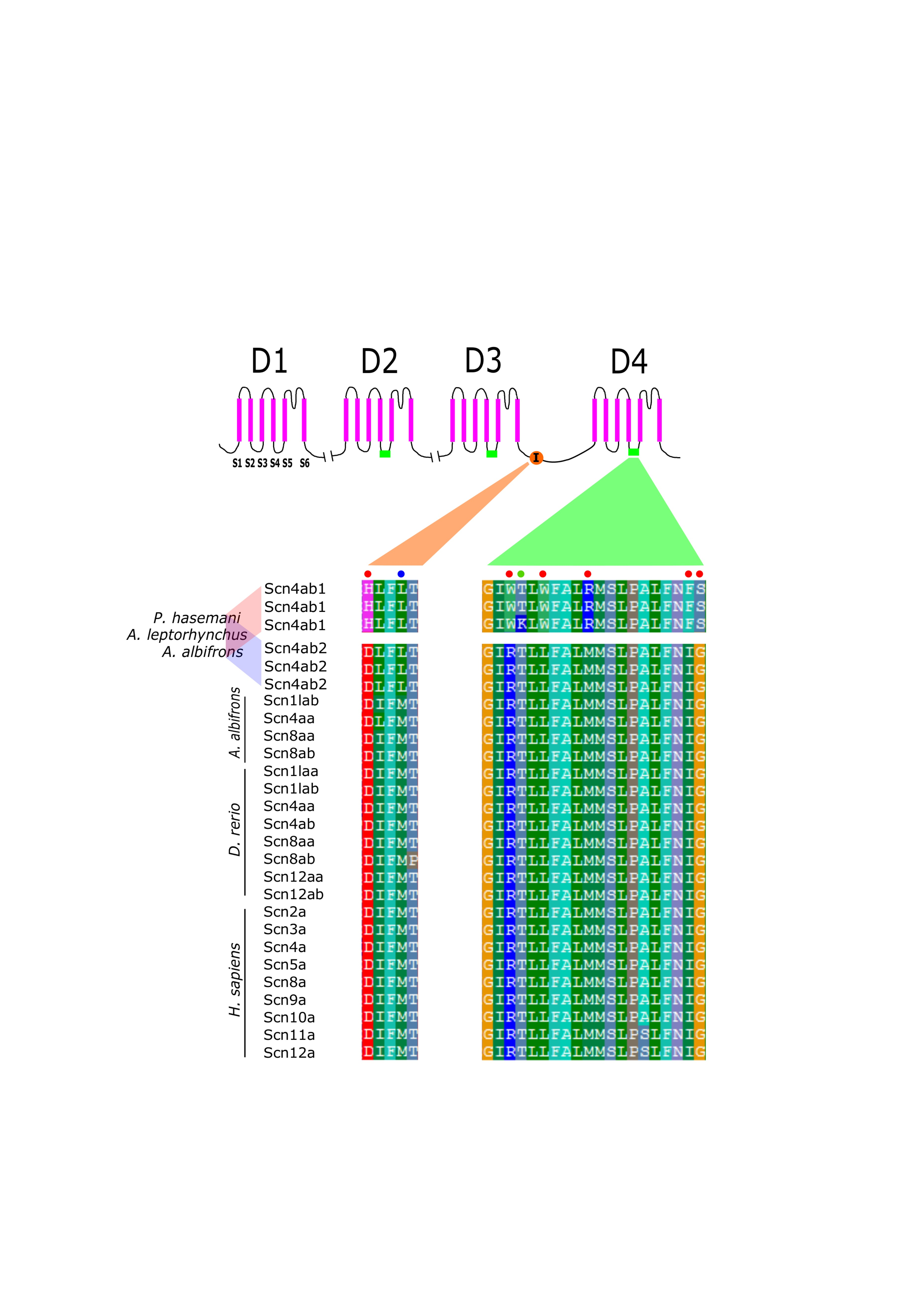

Supplement: S6 Fig — Some amino acid substitutions occur in all Apteronotid scn4ab channels (blue dot), some in all Apteronotini (red dots), and 1 only in A. albifrons (green dot). Nav, voltage-gated sodium. (TIF) [file pbio.2004892.s006.tif]

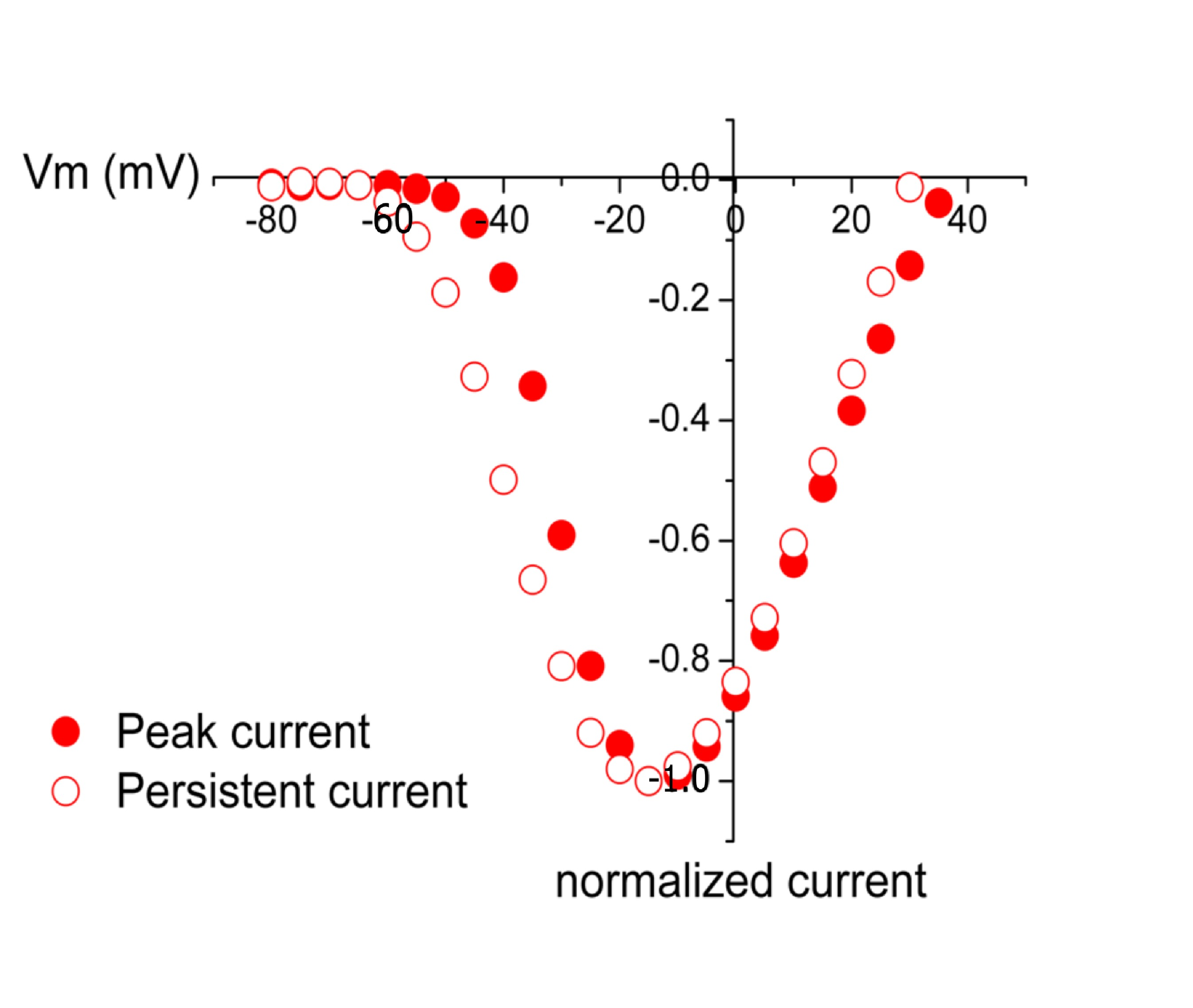

Supplement: S7 Fig — Note that the persistent current activates at a more negative voltage than the peak current and that it comprises a relatively greater fraction of the peak current in the range from −60 to −40 mV. Figure data included in S2 Data. (TIF) [file pbio.2004892.s007.tif]

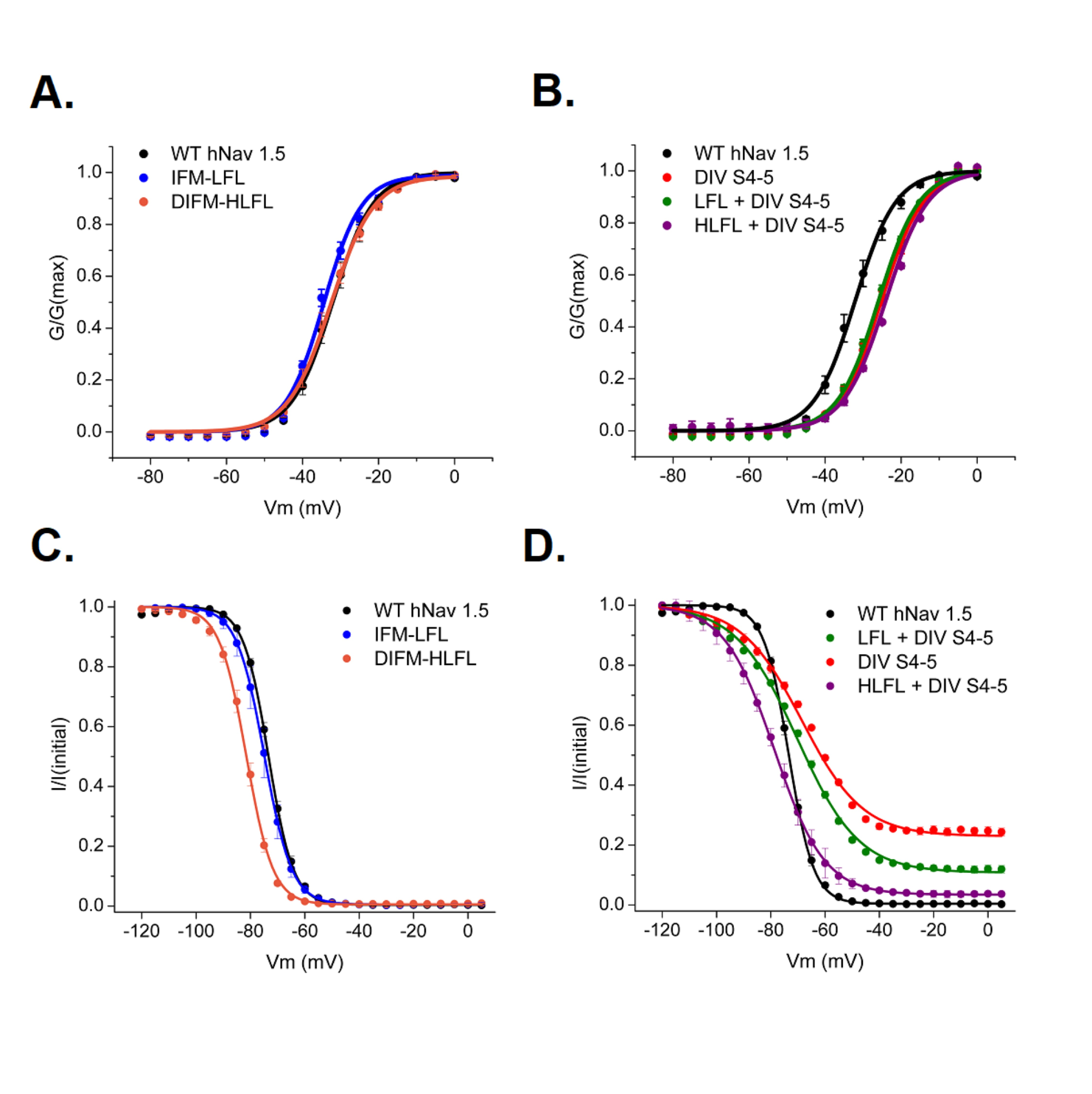

Supplement: S8 Fig — A. Inactivation particle substitutions (see Figs 3 and S6) did not affect channel activation. (B) Apteronitini-specific domain 4 S4–S5 substitutions were associated with a 7–10 mV depolarizing shift in channel activation either alone or in conjunction with substitutions in the inactivation particle. (C) Effects of Apteronotini (DIFM to HLFL) inactivation particle substitutions on steady-state inactivation. Xenopus oocytes expressing sodium channel variants were subjected to a 500-millisecond conditioning pulse at a given voltage, followed by a 1-millisecond step at −100 mV and a 20-millisecond test pulse at −20 mV. (D) Same as panel C but showing effects of Apteronotini domain 4 S4–S5 substitutions on steady-state inactivation alone and in conjunction with substitutions in the inactivation particle. Note the nonzero asymptotes for steady-state inactivation in the D4 S4–S5 variants. N ≥ 5 for each variant; quantification in S4 Table. Figure data included in S2 Data. D, aspartate; F, phenylalanine; H, histidine; I, isoleucine; M, methionine; Nav, voltage-gated sodium. (TIF) [file pbio.2004892.s008.tif]

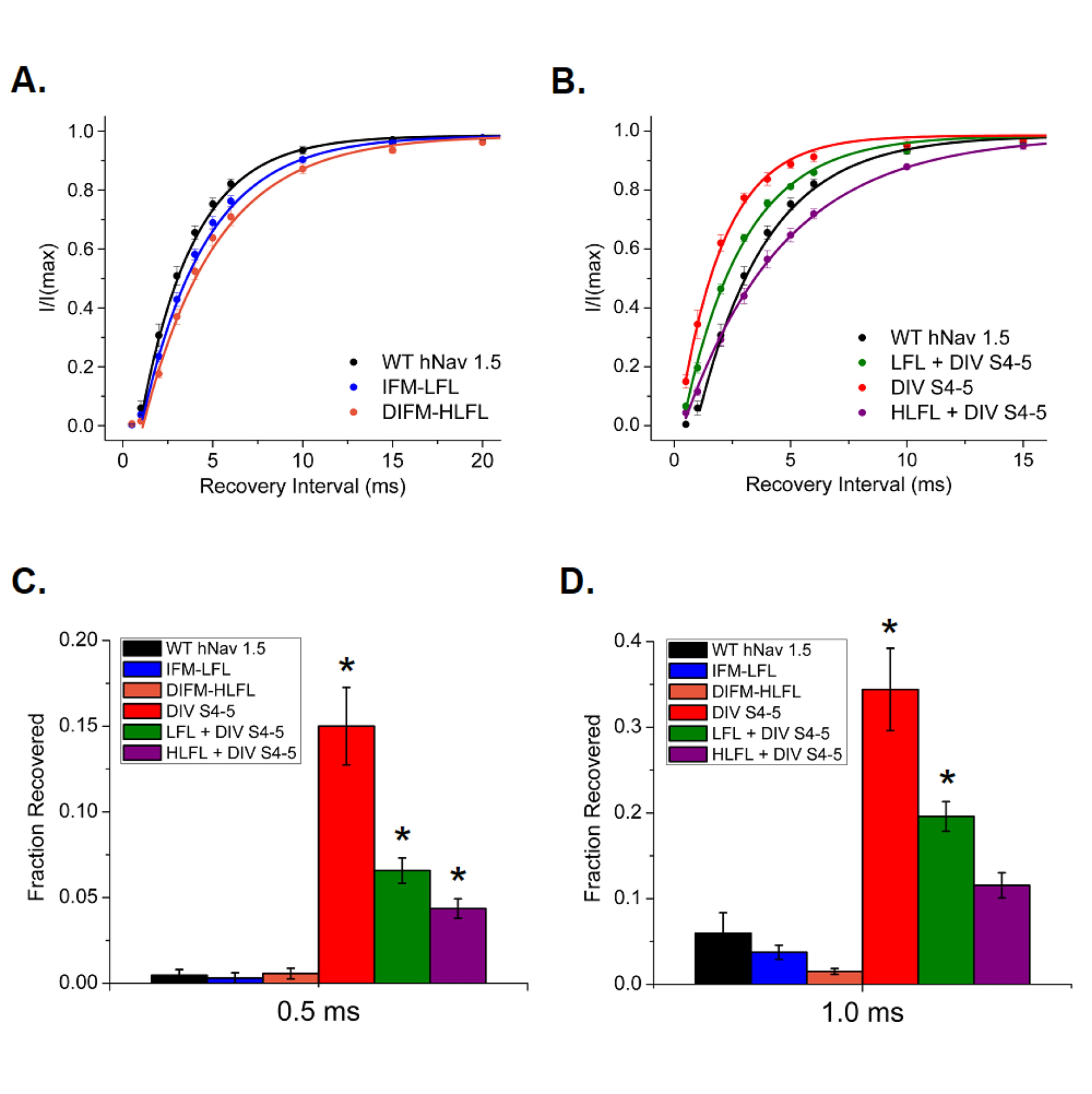

Supplement: S9 Fig — Sodium channel currents were activated via 20-millisecond pulses to −20 mV, which were separated by a recovery interval at −120 mV for a specified length of time. (A) Normalized fraction of current as a function of recovery interval for WT hNav1.5 and Apteronotinti inactivation particle substitutions. (B) Same as panel A but showing effects of Apteronotinti Nav1.4ab domain 4 S4–S5 substitutions on recovery from fast inactivation alone and in conjunction with knifefish substitutions in the inactivation particle. (C) Quantification of fraction of current recovered at the shortest recovery interval (0.5 milliseconds). Asterisk indicates variants with statistically significant differences (p < 0.01) as compared to WT hNav1.5. (D) Same as panel C but showing fraction recovered by 1.0 milliseconds. N ≥ 4 for each variant. In panel C and D, note that the domain 4 S4–S5 substitutions (red bar) increased the fraction of current recovered as compared to hNav1.5 (indicating faster recovery from inactivation). Inclusion of the inactivation particle substitutions (green and purple bars) reduced this effect. Figure data included in S2 Data. hNav, human voltage-gated sodium; WT, wild-type. (TIF) [file pbio.2004892.s009.tif]

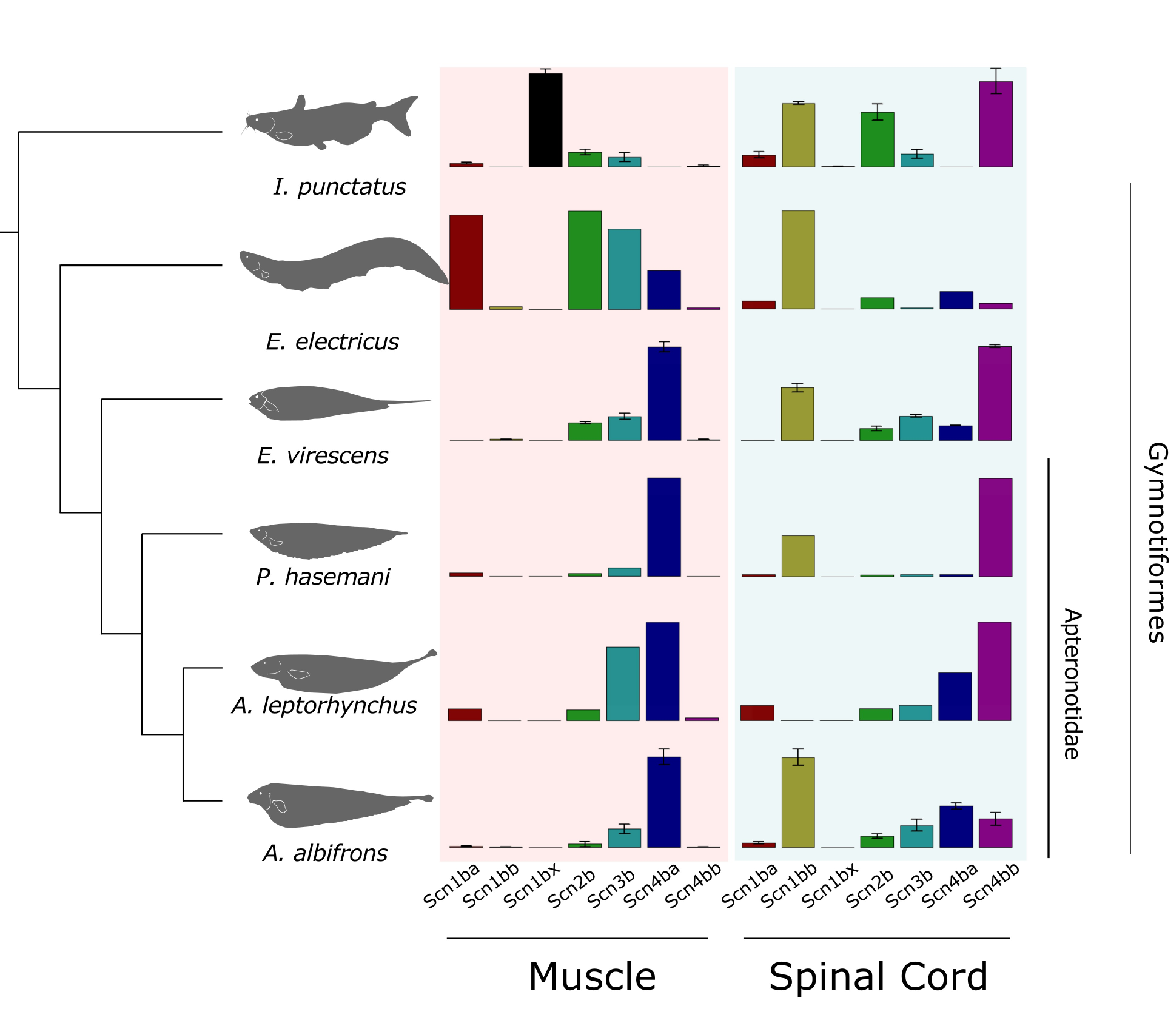

Supplement: S10 Fig — Beta subunits are known to modify the properties of Nav channels. Note that different subunits are expressed in the muscle and spinal cord of Apteronotids. Figure data included in S1 Data. Nav, voltage-gated sodium. (TIF) [file pbio.2004892.s010.tif]
