# Supplementary material for: Rapid evolution of a voltage-gated sodium channel gene in a lineage of electric fish leads to a persistent sodium current
Source: PLoS Biol. 2018 Mar 27;16(3):e2004892. doi: 10.1371/journal.pbio.2004892 (PMC5870949; doi:10.1371/journal.pbio.2004892)
Supplement: S1 Table — (DOCX) [file pbio.2004892.s011.docx]

| **Table 1. Gene accessions for Gene tree Analysis** | | |
| --- | --- | --- |
| Species | Gene | Genbank/Ensemble Accessions |
| *Homo sapiens* | *Scn4a* | NM_000334.4 |
| *Sternopygus macrurus* | *Scn4ab* | AF378139.2 |
| *Electrophorus electricus* | *Scn4ab* | GU362053.1 |
| *Eigenmannia virescens* | *Scn4ab* | GU362054.1 |
| *Danio rerio* | *Scn4ab* | NM_001045065.1 |
| *Gymnotus cylindricus* | *Scn4ab* | GU362052.1 |
| *Astyanax mexicanus* | *Scn4ab* | XP_007241580.1 |
| *Rhamphichthys marmoratus* | *Scn4ab* | GU362049.1 |
| *Brachyhypopomus pinnicaudatus* | *Scn4ab* | GU362050.1 |
| *Poecilia formosa* | *Scn4ab* | XM_016677957.1 |
| *Steatogenys elegans* | *Scn4ab* | GU362051.1 |
| *Gadus morhua* | *Scn4ab* | ENSGMOT00000010937.1 |
| *Oryzias latipes* | *Scn4ab* | ENSORLT00000024364.1 |
| *Xiphophorus maculatus* | *Scn4ab* | ENSXMAT00000012940.1 |
| *Ictalurus punctatus* | *Scn4ab* | XM_017481721.1 |
| *Adontosternarchus devenanzii* | *Scn4ab* | MG969486 |
| *Apteronotus bonapartii* | *Scn4ab* | MG969487 |
| *A. albifrons* | *scn4aa* | MG969479 |
| *A. albifrons* | *scn4ab1* | MG969480 |
| *A. albifrons* | *scn4ab2* | MG969481 |
| *A. leptorhynchus* | *scn4ab1* | MG969483 |
| *A. leptorhynchus* | *scn4ab2* | MG969482 |
| *P. hasemani* | *scn4ab1* | MG969485 |
| *P. hasemani* | *scn4ab2* | MG969484 |
