# Supplementary material for: Rapid evolution of a voltage-gated sodium channel gene in a lineage of electric fish leads to a persistent sodium current
Source: PLoS Biol. 2018 Mar 27;16(3):e2004892. doi: 10.1371/journal.pbio.2004892 (PMC5870949; doi:10.1371/journal.pbio.2004892)
Supplement: S2 Table — (DOCX) [file pbio.2004892.s012.docx]

|  |  |  |  |  |  |
| --- | --- | --- | --- | --- | --- |
| **Table 2. Accessions for genes used for Chronogram** | | | | | |
| Genus | Species | Individual | CytB | RAG2 | COI |
| *Orthosternarchus* | *tamandua* | 1 | KT932231 | KT932268 | KT932165 |
| *Sternarchorhamphus* | *muelleri* | 1 | N/A | KT932272 | KT932183 |
| *Sternarchorhamphus* | *muelleri* | 2 | N/A | KT932263 | KT932184 |
| *Sternarchorhamphus* | *muelleri* | 3 | KT932235 | N/A | KT932185 |
| *Sternarchorhamphus* | *muelleri* | 4 | N/A | KT932298 | KT932198 |
| *Sternarchorhamphus* | *muelleri* | 5 | N/A | KT932291 | KT932182 |
| *Adontosternarchus* | *clarkae* | 1 | KT932201 | KT932254 | KT932134 |
| *Adontosternarchus* | *clarkae* | 2 | N/A | N/A | KT932131 |
| *Adontosternarchus* | *clarkae* | 3 | N/A | KT932299 | KT932132 |
| *Adontosternarchus* | *clarkae* | 4 | N/A | KT932302 | KT932133 |
| *Adontosternarchus* | *devenanzii* | 1 | KT932226 | N/A | KT932135 |
| *Adontosternarchus* | *devenanzii* | 2 | N/A | N/A | KT932136 |
| *Adontosternarchus* | *nebulosus* | 1 | KT932243 | N/A | KT932137 |
| *Adontosternarchus* | *nebulosus* | 2 | N/A | N/A | KT932138 |
| *Adontosternarchus* | *balaenops* | 1 | KT932245 | KT932290 | KT932197 |
| *Adontosternarchus* | *balaenops* | 2 | KT932207 | KT932288 | KT932130 |
| *Adontosternarchus* | *sachi* | 1 | N/A | KT932304 | KT932139 |
| *Platyurosternarchus* | *macrostomus* | 1 | KT932229 | KT932270 | KT932167 |
| *Platyurosternarchus* | *macrostomus* | 2 | KT932232 | KT932252 | KT932168 |
| *Platyurosternarchus* | *macrostomus* | 3 | N/A | KT932269 | N/A |
| *Sternarchorhynchus* | *roseni* | 1 | KT932224 | N/A | KT932193 |
| *Sternarchorhynchus* | *galibi* | 1 | KT932223 | KT932287 | KT932186 |
| *Sternarchorhynchus* | *mormyrus* | 1 | KT932236 | KT932300 | KT932188 |
| *Sternarchorhynchus* | *mormyrus* | 2 | KT932225 | KT932303 | N/A |
| *Sternarchorhynchus* | *oxyrhynchus* | 1 | KT932205 | KT932265 | KT932189 |
| *Sternarchorhynchus* | *oxyrhynchus* | 2 | KT932206 | KT932264 | KT932190 |
| *Sternarchorhynchus* | *oxyrhynchus* | 3 | N/A | N/A | KT932191 |
| *Sternarchorhynchus* | *oxyrhynchus* | 4 | N/A | KT932273 | KT932192 |
| *Sternarchorhynchus* | *oxyrhynchus* | 5 | N/A | N/A | KT932195 |
| *Sternarchorhynchus* | *oxyrhynchus* | 6 | N/A | KT932286 | KT932196 |
| *Sternarchorhynchus* | *mesensis* | 1 | N/A | KT932271 | KT932187 |
| *Sternarchogiton* | *preto* | 1 | KT932199 | KT932281 | KT932180 |
| *Sternarchogiton* | *preto* | 2 | KT932215 | KT932277 | KT932181 |
| *Sternarchogiton* | *porcinum* | 1 | KT932215 | KT932257 | KT932178 |
| *Sternarchogiton* | *porcinum* | 2 | N/A | KT932285 | KT932179 |
| *Sternarchogiton* | *nattereri* | 1 | KT932233 | KT932258 | KT932176 |
| *Sternarchogiton* | *nattereri* | 2 | KT932218 | KT932283 | KT932177 |
| *Compsaraia* | *compsus* | 1 | KT932248 | KT932292 | KT932160 |
| *Compsaraia* | *compsus* | 2 | KT932212 | KT932259 | KT932162 |
| *Compsaraia* | *samueli* | 1 | KT932246 | KT932255 | KT932163 |
| *Compsaraia* | *samueli* | 2 | KT932247 | KT932260 | KT932164 |
| *Compsaraia* | *samueli* | 3 | N/A | N/A | KT932194 |
| *Compsaraia* | *samueli* | 4 | N/A | N/A | KT932161 |
| *Sternarchella* | *calhamazon* | 1 | KT932234 | KT932284 | KT932172 |
| *Sternarchella* | *calhamazon* | 2 | N/A | KT932296 | KT932173 |
| *Sternarchella* | *calhamazon* | 3 | N/A | N/A | KT932175 |
| *Sternarchella* | *schotii* | 1 | N/A | N/A | KT932174 |
| *Magosternarchus* | *raptor* | 1 | KT932237 | KT932253 | N/A |
| *Magosternarchus* | *raptor* | 2 | KT932241 | KT932301 | N/A |
| *Apteronotus* | *bonapartii* | 1 | KT932222 | KT932289 | KT932144 |
| *Apteronotus* | *bonapartii* | 2 | KT932228 | N/A | KT932145 |
| *Apteronotus* | *bonapartii* | 3 | N/A | N/A | N/A |
| *Apteronotus* | *apurensis* | 1 | KT932211 | KT932275 | KT932143 |
| *Porotergus* | *gimbeli* | 1 | KT932213 | KT932250 | KT932169 |
| *Porotergus* | *gimbeli* | 2 | KT932203 | KT932251 | N/A |
| *Porotergus* | *gimbeli* | 3 | KT932204 | KT932256 | KT932170 |
| *Porotergus* | *gymnotus* | 1 | KT932238 | KT932305 | KT932171 |
| *Porotergus* | *gymnotus* | 2 | N/A | KT932278 | KT932159 |
| *Parapteronotus* | *hasemani* | 1 | KT932202 | KT932261 | N/A |
| *Parapteronotus* | *hasemani* | 2 | KT932200 | KT932262 | N/A |
| *Parapteronotus* | *hasemani* | 3 | N/A | N/A | KT932166 |
| *Apteronotus* | *albifrons* | 1 | KT932208 | KT932293 | KT932140 |
| *Apteronotus* | *albifrons* | 2 | KT932220 | KT932295 | N/A |
| *Apteronotus* | *albifrons* | 3 | KT932227 | N/A | N/A |
| *Apteronotus* | *albifrons* | 4 | KT932219 | N/A | N/A |
| *Apteronotus* | *albifrons* | 5 | KT932221 | N/A | N/A |
| *Apteronotus* | *albifrons* | 6 | KT932209 | KT932274 | KT932141 |
| *Apteronotus* | *albifrons* | 7 | KT932242 | N/A | KT932142 |
| *Apteronotus* | *magdalenensis* | 1 | N/A | KT932282 | KT932154 |
| *Apteronotus* | *magdalenensis* | 2 | KT932249 | KT932266 | KT932155 |
| *Apteronotus* | *galvisi* | 1 | N/A | N/A | KT932147 |
| *Apteronotus* | *galvisi* | 2 | N/A | N/A | KT932148 |
| *Apteronotus* | *galvisi* | 3 | KT932239 | KT932276 | KT932149 |
| *Apteronotus* | *anu* | 1 | KT932230 | N/A | N/A |
| *Apteronotus* | *anu* | 2 | KT932210 | KT932297 | KT932150 |
| *Apteronotus* | *anu* | 3 | N/A | N/A | KT932151 |
| *Apteronotus* | *anu* | 4 | N/A | N/A | KT932152 |
| *Apteronotus* | *anu* | 5 | N/A | N/A | KT932153 |
| *Apteronotus* | *eschmeyeri* | 1 | KT932216 | N/A | N/A |
| *Apteronotus* | *eschmeyeri* | 2 | KT932244 | N/A | KT932146 |
| *Apteronotus* | *mariae* | 1 | KT932240 | KT932280 | KT932156 |
| *Apteronotus* | *mariae* | 2 | KT932217 | N/A | KT932157 |
| *Apteronotus* | *rostratus* | 1 | N/A | KT932267 | KT932158 |
