# Supplementary material for: Rapid evolution of a voltage-gated sodium channel gene in a lineage of electric fish leads to a persistent sodium current
Source: PLoS Biol. 2018 Mar 27;16(3):e2004892. doi: 10.1371/journal.pbio.2004892 (PMC5870949; doi:10.1371/journal.pbio.2004892)
Supplement: S3 Table — (DOCX) [file pbio.2004892.s013.docx]

| **Table 3. Supplemental Sequences for Chronogram** | | | |
| --- | --- | --- | --- |
| Species | COI | CytB | RAG2 |
| *Adontosternarchus devenanzii* | KR491532 | KR491639 | KR491811 |
| *Adontosternarchus nebulosus* | KR491530 | KR491641 | KR491812 |
| *Apteronotus leptorhynchus* | KR491533 | KR491644 | KR491818 |
| *Platyurosternarchus crypticus* | KR491539 | KR491649 | KR491819 |
| *Sternarchella terminalis* | KR491542 | KR491670 | KR491834 |
| *Serrasalmus maculatus* | GU701633 | HQ289574 | HQ289382 |
| *Danio rerio* | FJ459455 | JN234180 | AY804070 |
| *Clarias batrachus* | KF604656 | DQ119486 | DQ492408 |
| *Ictalurus punctatus* | HQ024941 | AB045119 | DQ492398 |
| *Gymnotus carapo* | KF533345 | GQ862601 | GQ862546 |
| *Sternopygus macrurus* | KT932129 | KF533300 | KT932294 |
| *Eigenmannia virescens* | GU702089 | KF533297 | KF533320 |
