# Supplementary material for: Rapid evolution of a voltage-gated sodium channel gene in a lineage of electric fish leads to a persistent sodium current
Source: PLoS Biol. 2018 Mar 27;16(3):e2004892. doi: 10.1371/journal.pbio.2004892 (PMC5870949; doi:10.1371/journal.pbio.2004892)
Supplement: S4 Table — (DOCX) [file pbio.2004892.s014.docx]

S4 Table

|  | Activation | | | | | Steady State Inactivation | | | | | | |
| --- | --- | --- | --- | --- | --- | --- | --- | --- | --- | --- | --- | --- |
|  | V_1/2_ | STD | dx | STD | N | V_1/2_ | STD | dx | STD | Asympt.  Fraction | STD | N |
| WT hNa_v_ 1.5 | -32.5 | 2.8 | 5.20 | 0.48 | 6 | -73.2 | 1.1 | 4.50 | 0.19 | 0.005 | 0.009 | 5 |
| IFM-LFL | -35.1 | 1.6 | 5.02 | 0.53 | 5 | -75.0 | 4.1 | 4.50 | 0.15 | 0.007 | 0.010 | 6 |
| DIFM-HLFL | -32.9 | 2.5 | 5.76 | 0.27 | 5 | **-81.3** | 1.7 | 4.64 | 0.25 | 0.008 | 0.007 | 5 |
| D4 S4-S5 | **-24.9** | 1.9 | **6.07** | 0.34 | 7 | **-67.5** | 2.6 | **10.49** | 0.72 | **0.247** | 0.043 | 6 |
| LFL+D4 S4-S5 | **-25.9** | 1.1 | **5.91** | 0.16 | 6 | **-69.7** | 1.6 | **10.89** | 0.55 | **0.120** | 0.028 | 5 |
| HLFL+D4 S4-S5 | **-22.5** | 0.2 | 5.75 | 0.24 | 5 | **-78.0** | 2.2 | **11.01** | 1.11 | **0.037** | 0.003 | 5 |

BOLD indicates p<0.01 with respect to WT hNa_v_ 1.5

STD = standard deviation

Asympt. Fraction is the fraction of current that was resilient to SSI, even at most depolarized potentials.
